# CBX2 is required to stabilize the testis pathway by repressing Wnt signaling

**DOI:** 10.1371/journal.pgen.1007895

**Published:** 2019-05-22

**Authors:** S. Alexandra Garcia-Moreno, Yi-Tzu Lin, Christopher R. Futtner, Isabella M. Salamone, Blanche Capel, Danielle M. Maatouk

**Affiliations:** 1 Department of Obstetrics and Gynecology, Northwestern University, Chicago, Illinois, United States of America; 2 Department of Cell Biology, Duke University Medical Center, Durham, North Carolina, United States of America; MRC Human Genetics Unit, UNITED KINGDOM

## Abstract

XX and XY fetal gonads are initially bipotential, poised between the ovary and testis fate. Multiple lines of evidence suggest that commitment to testis fate requires the repression of genes associated with ovary fate. It was previously shown that loss of CBX2, the subunit of the Polycomb Repressive Complex 1 (PRC1) that binds H3K27me3 and mediates silencing, leads to ovary development in XY mice and humans. While it had been proposed that CBX2 is an activator of the testis-determining gene *Sry*, we investigated the alternative possibility that CBX2 has a direct role as a repressor of the antagonistic ovary-promoting pathway. To investigate this possibility, we developed a quantitative genome-wide profile of the repressive histone mark H3K27me3 and its active counterpart H3K4me3 in isolated XY and XX gonadal supporting cells before and after sex determination. We show that testis and ovary sex-determining (SD) genes are bivalent before sex determination, providing insight into how the bipotential state of the gonad is established at the epigenetic level. After sex determination, many SD genes of the alternate pathway remain bivalent, possibly contributing to the ability of these cells to transdifferentiate even in adults. The finding that many genes in the Wnt signaling pathway were targeted for H3K27me3-mediated repression in Sertoli cells led us to test whether deletion of *Wnt4* could rescue testis development in *Cbx2* mutants. We show that *Sry* expression and testis development were rescued in XY *Cbx2*^*-/-*^*;Wnt4*^*-/-*^ mice. Furthermore, we show that CBX2 directly binds the downstream Wnt signaler *Lef1*, an ovary-promoting gene that remains bivalent in Sertoli cells. Our results suggest that stabilization of the testis fate requires CBX2-mediated repression of bivalent ovary-determining genes, which would otherwise block testis development.

## Author summary

During development, the bipotential fetal gonad can commit to the testis fate or to the ovary fate. Mutation of the epigenetic regulator CBX2 leads to ovary development in XY embryos, suggesting a critical role for chromatin remodeling during sex determination. However, the epigenetic mechanisms that regulate the testis vs. ovary cell-fate decision in the mammalian bipotential gonad are poorly understood. In this study, we developed a genome-wide profile of two histone modifications that play critical roles during development: H3K27me3 (repressive) and H3K4me3 (active). We find that sex-determining genes that are initially co-expressed in XX and XY bipotential gonads are bivalent (marked by both H3K4me3 and H3K27me3) prior to sex determination, poised to engage either the testis or ovary fate. Remarkably, after sex determination, repressed genes that promote the alternate fate remain bivalent. We show that stabilization of the testis fate requires CBX2-mediated repression of bivalent ovary-determining genes, which would otherwise block testis development. Our study provides insight into how the bipotential state of the gonad is established at the epigenetic level, and how the testis fate is stabilized by repression of the ovary fate during sex determination.

## Introduction

Gonadal sex determination, the process by which the bipotential fetal gonad initiates development as either a testis or an ovary, is the first critical step in the development of sexually dimorphic internal and external reproductive organs. Sex determination initiates with a binary cell fate decision within a single somatic cell lineage of the gonad, known as the supporting cell lineage [1, 2]. Cells of this lineage are initially held in a bipotential state in the early fetal gonad, poised between testis and ovary fate. In most mammalian species, including mice and humans, expression of the Y-encoded transcription factor *Sex-determining Region Y* (*Sry*) is required to direct the testis fate of supporting cells [3–5]. In mice, *Sry* is transiently expressed in XY progenitor cells from ~E10.5-E12.5, soon after the gonad is first formed [6, 7]. *Sry’s* primary function is to upregulate its downstream target *Sox9* [8]. *Sox9* upregulation and subsequent maintenance through *Fgf9* leads to Sertoli cell differentiation and establishment of the testis pathway [9]. In the absence of a Y chromosome, the canonical Wnt signaling molecules *Wnt4* and *Rspo1* become upregulated in XX progenitor cells (Vainio, 1999, Chassot, 2008). The subsequent downstream stabilization of β-catenin [10] together with upregulation of other transcription factors such as *Foxl2* [11], leads to the differentiation of pregranulosa cells in the ovary.

Importantly, upregulation of either Sertoli- or pregranulosa-promoting pathways is accompanied by mutually antagonistic mechanisms, which are critical for repressing the alternate pathway at the time of sex determination [9, 12]. Mapping the up- or down-regulation of each gene in the XX and XY gonad between E11.0 and E12.0 revealed that many genes associated with the ovarian pathway became ovary-specific by down-regulation in the XY gonad, while a smaller group of testis genes became testis-specific by down-regulation in the XX gonad [13, 14]. This data established the importance of gene repression in the initiation of testis (or ovary) development.

Interestingly, repression of the alternative fate is actively maintained throughout adulthood. Evidence for this came from a conditional deletion of *Foxl2* in post-natal granulosa cells, which led to their transdifferentiation towards a Sertoli-like state, and reorganization of the ovary into testicular tissue [15]. Conversely, conditional deletion of the testis-determining gene *Dmrt1* in post-natal testes led to transdifferentiation of Sertoli cells into granulosa cells accompanied by ovarian reorganization [16]. This ability to transdifferentiate to the alternate fate highlights both the highly plastic nature of sex determination and the importance of repressive mechanisms that can stably transmit the initial fate decision long after commitment in fetal life.

There is increasing evidence that cell differentiation is epigenetically regulated. During sex determination, the chromatin modifiers GLP-G9A/JMJD1A and CBP/P300 regulate the repressive H3K9me2 mark and the active H3K27ac mark at the *Sry* promoter [17–19]. However, the epigenetic mechanisms that regulate the testis vs. ovary fate at a genome-wide level remain poorly understood. In embryonic stem cells and other pluripotent systems, the Polycomb group (PcG) of epigenetic factors have emerged as critical regulators of cell fate specification by maintaining both pluripotency and cell identity through sustained repression of differentiation programs. The PcG proteins assemble into two multi-protein complexes, Polycomb Repressive Complex 1 and 2 (PRC1 and PRC2), which can functionally cooperate to repress target genes [20]. PRC2 establishes H3K27me3 through its catalytic component EZH1/2 [21, 22]. PRC1 can form either canonical (cPRC1) or non-canonical (ncPRC1) sub-complexes depending on the composition of its subunits. While cPRC1 contains a CBX subunit that binds H3K27me3 and promotes chromatin compaction [23], ncPRC1 complexes lack CBX proteins and their recruitment to chromatin is independent of H3K27me3 [24, 25].

The PcG proteins often work alongside the Trithorax group of proteins (TrxG), which have the opposite role of maintaining transcriptional expression through the deposition of H3K4me3 at active promoters [26]. The co-occurrence of an active and a repressive histone modification at the promoter of developmental genes maintains these so-called “bivalent” genes in a repressed or lowly expressed state, poised for activation upon reception of a developmental signal [27–29]. Hence, the cooperative function of PcG and TrxG adds a layer of regulation by fine-tuning the timing of gene expression and stabilizing cell fate commitment throughout cell division. While CpG islands, transcription factors, and non-coding RNAs appear to play a major role in the establishment of bivalent loci, the exact mechanisms that recruit the PcG and TrxG complexes to specific targets remain elusive [30].

It was previously reported that loss of the cPRC1 subunit CBX2 leads to ovary development in XY mice and humans, suggesting that cPRC1 is required for commitment to the testis fate. Although it was originally proposed that *Cbx2* acts as a direct activator of *Sry*, here we show that XY *Cbx2*^*-/-*^ ovaries have some SOX9-positive cells, implying that *Sry* was expressed in at least some cells in the absence of *Cbx2*. Therefore, we considered the possibility that sex reversal in XY *Cbx2* mutants is caused by a failure to repress the ovarian pathway, rather than a failure to upregulate *Sry*. To identify targets of PcG and TrxG, we compared the genome-wide profiles of H3K27me3 and H3K4me3 in the supporting cell lineage of the gonad at the bipotential stage (E10.5) and after sex determination has occurred (E13.5). We found that key sex-determining (SD) genes are bivalent prior to sex determination, providing insight into how the bipotential state of gonadal progenitor cells is established. Surprisingly, repressed SD genes remain bivalent after sex-determination and, at least in Sertoli cells, bivalency is retained even in adulthood, possibly contributing to the highly plastic nature of the gonad long after the initial fate commitment. This finding also suggests that maintenance of H3K27me3 at ovary-promoting genes throughout sex determination is critical for stabilization of the testis fate. Our finding that the Wnt signaling pathway is a primary target of PcG repression in testes led us to test the possibility that loss of Wnt signaling could rescue testis development in *Cbx2* mutants. We show that *Sry* expression and testis development were rescued in XY *Cbx2*^*-/-*^*;Wnt4*^*-/-*^ double knockout mice, suggesting that at least in the absence of *Wnt4*, *Cbx2* is not required to activate *Sry* as previously proposed. Furthermore, we show that CBX2 targets the bivalent promoter of the downstream Wnt signaler *Lef1* in embryonic and adult testes, supporting a role for CBX2 as a stabilizer of repression at bivalent genes that would otherwise drive ovary fate.

## Results

### XY *Cbx2*^*-/-*^ supporting cells fail to repress the ovarian pathway

**Fig 1:**
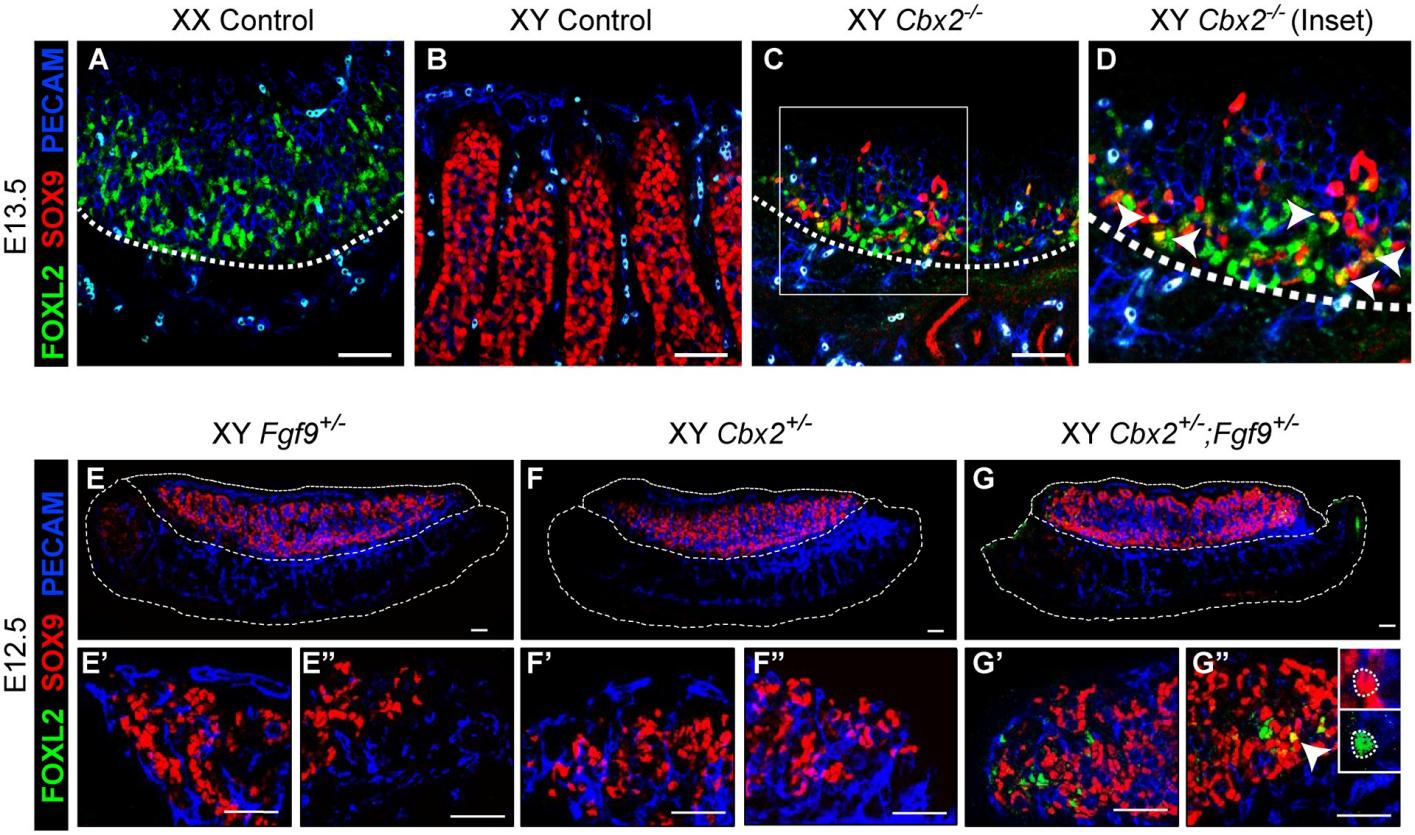
Loss of *Cbx2* in XY cells leads to upregulation of the ovary pathway. E13.5 (A-D) and E12.5 (E-G”) are stained with the pregranulosa cell marker FOXL2 (green), Sertoli cell marker SOX9 (red), and germ cell and vasculature marker PECAM (blue). WT XX gonads have FOXL2-expressing pregranulosa cells (A), whereas WT XY gonads have SOX9-expressing Sertoli cells (red), which are organized around germ cells forming testis cords (B). (C&D) Loss of *Cbx2* in E13.5 XY gonads leads to reduction of SOX9+ Sertoli cells (red) and gain of FOXL2+ pregranulosa cells (green). Some individual cells express both markers (yellow, arrowheads in D). Testis cords are lost and the morphology resembles WT XX gonads (A). XY gonads of single heterozygotes show no evidence of FOXL2 expression (E, F). The anterior (left, eg. E’) and posterior (right, eg. E”) poles of each gonad are enlarged in the bottom row. Gonads of *Cbx2;Fgf9* double heterozygous mice (G) have FOXL2+ pregranulosa cells at the gonadal poles (G’ and G”). Some individual cells express both markers (yellow, arrowhead). A magnified view in single channels of the cell indicated by the arrowhead are in the upper-right corner (G”). Scale bars, 50μm.

It was previously shown that loss of the cPRC1 subunit CBX2 leads to complete XY sex reversal in mice and humans, which was proposed to result from a failure to upregulate *Sry* and *Sox9* expression [31–33]. Although it was proposed that CBX2 activates *Sry*, it remained unclear whether CBX2 is a direct mediator of *Sry*, or whether it functions indirectly by repressing an antagonist of *Sry*. We found that at E13.5, XY *Cbx2*^*-/-*^ mutant gonads have both FOXL2- and SOX9-expressing cells, with fewer SOX9-expressing cells than in wild-type XY gonads (Fig 1A–1D). In addition, we found some individual cells that express both markers simultaneously (arrows in Fig 1D). Testis cords that typically characterize this stage of testis development (Fig 1B) were completely absent (Fig 1C), and *Cbx2*^*-/-*^ gonads developed as hypoplastic ovaries as reported previously [33]. As activation of *SOX9* typically requires SRY in a wild-type background, this phenotype suggested to us that sex reversal is due to a failure to repress the ovary pathway in XY cells, rather than a failure to activate *Sry* and *Sox9*.

This finding is consistent with several previous lines of work that showed that the testis and ovary pathways are mutually antagonistic. For example, activation of Wnt signaling in the XY gonad can suppress testis development [10]. In XY gonads, Wnt signaling is repressed (at least partly) by the E3 ubiquitin-protein ligase ZNRF3 [34] and the testis-determining FGF9 [9, 12]. To investigate whether CBX2 synergizes with FGF9 to repress the ovary pathway, we crossed *Fgf9* and *Cbx2* mutants. Although homozygous loss of either *Cbx2* or *Fgf9* leads to complete XY sex reversal [31, 35], gonads with heterozygous loss of either gene develop normally (Fig 1E–1G”). However, gonads of *Cbx2*^*+/-*^*;Fgf9*^*+/-*^ double heterozygotes show partial XY sex reversal at the gonadal poles (Fig 1G”), similar to other models of partial sex reversal [12, 36]. Furthermore, XY double heterozygotes have cells with simultaneous expression of FOXL2 and SOX9, similar to XY *Cbx2*^*-/-*^ gonads (arrow in Fig 1G”). Taken together, these experiments suggest that *Cbx2* functions as a repressor of the ovary fate during testis development.

### *In vivo* chromatin profiling of gonadal supporting cells

Transcriptional profiling of supporting cells throughout sex determination revealed that at the earliest stages of *Sry* expression, a large network of testis- or ovary-promoting signaling pathways coexist [13]. At this stage, more pregranulosa-promoting genes are expressed than Sertoli-promoting genes [14]. Commitment to the ovary fate depends on continued activation of pregranulosa-promoting genes with little change to Sertoli-promoting genes. In contrast, initiation of the testis pathway requires both upregulation of the testis pathway and simultaneous repression of the ovary pathway [13].

**Fig 2:**
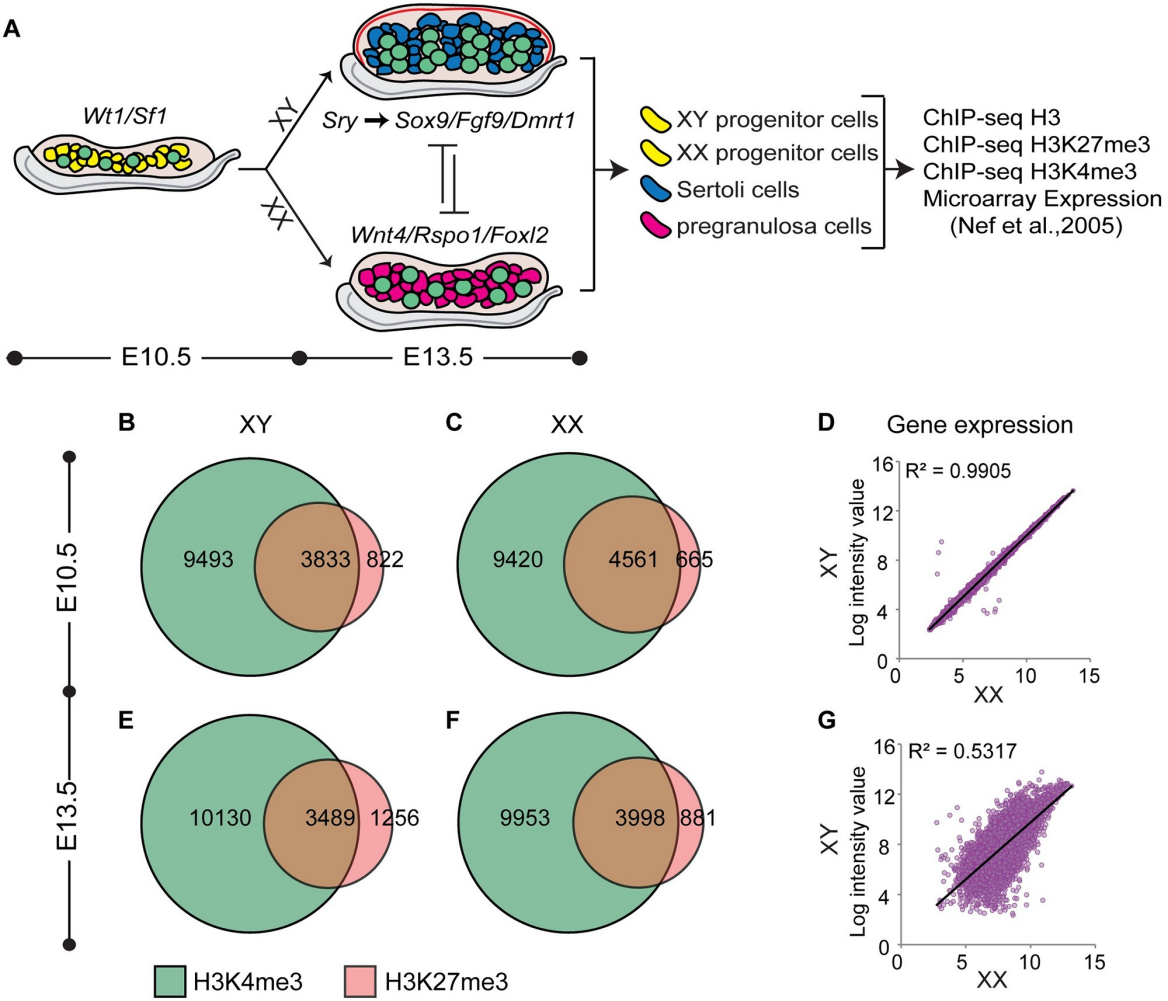
Numbers of bivalent promoters decline as expression becomes sexually dimorphic. (A) An overview of sex determination and work flow. Briefly, supporting progenitor cells (yellow) are bipotential and indistinguishable between XX and XY gonads. Expression of *Sry* directs Sertoli cell differentiation in the testis through upregulation of testis-determining genes such as *Sox9*, *Fgf9* and *Dmrt1*. Absence of *Sry* leads to upregulation of ovary-determining genes *Wnt4*, *Rspo1*, and *Foxl2*, directing differentiation of pregranulosa cells in the ovary. XY and XX progenitor cells, Sertoli cells, and pregranulosa cells were FACS-purified and submitted to ChIP-seq for H3, H3K4me3 and H3K27me3. Further analysis made use of microarray expression data from E10.5 and E13.5 purified supporting cells [41]. (B-C and E-F) Venn diagrams depicting number of promoters marked by H3K27me3 (red) and H3K4me3 (green) in XX and XY supporting cells at E10.5 (B&C) and E13.5 (E&F). (D&G) Linear correlation between expression profiles of XY (y axis) and XX (x axis) progenitor cells at E10.5 (D), and at E13.5 (G) (expression profiles from [41]).

Based on the finding that *Cbx2* mutants do not efficiently silence the ovary pathway (Fig 1), and the fact that CBX2 is part of a large epigenetic complex that targets hundreds of genes, we speculated that the PcG proteins have a widespread repressive role during XY sex determination to establish H3K27me3 silencing marks at genes associated with the ovary pathway. The PcG proteins functionally cooperate with TrxG proteins, which have the opposite role of maintaining transcriptional expression through the deposition of H3K4me3 [26–29]. To explore the chromatin landscape for these histone marks in XY and XX cells before and after sex determination, we performed ChIP-seq for H3K27me3, H3K4me3 and for Histone 3 (H3) as a means of normalizing across populations [37]. ChIP-seq was performed in FACS (fluorescence activated cell sorted)-purified XY and XX bipotential progenitor cells from E10.5 gonads of *Sf1-eGFP* transgenic mice [38], committed Sertoli cells from E13.5 gonads of *Sox9-CFP* transgenic mice [39], and committed pregranulosa cells from gonads of E13.5 *TESCO-mutant (TESMS)* -*CFP* transgenic mice [40] (Fig 2A). Two independent replicates were performed (each replicate contained pooled cells from multiple embryos), and results were further validated by ChIP-qPCR (S1 Fig).

ChIP-seq revealed that the largest subset of promoters in all cell types were marked only by H3K4me3 (42–45%), whereas a smaller subset was marked by H3K27me3, the majority of which also overlapped with H3K4me3 (e.g. bivalent promoters) (Fig 2B–2F and S2A Fig). To investigate the association between these histone modifications and transcriptional activity in the supporting cell lineage, we made use of the microarray gene expression dataset performed by Nef et al. 2005 [41] in FACS-purified XY and XX supporting cells from *Sf1-eGFP* transgenic mice at E10.5 and E13.5 [38]. As expected, we found that the average transcriptional level of promoters marked only by H3K4me3 is significantly higher than the transcriptional level from bivalent promoters and promoters marked only by H3K27me3 (S2B Fig). The difference in the average transcriptional level of genes with bivalent promoters versus those marked only by H3K27me3 is of no or low significance, consistent with previous observations that bivalent genes are expressed at low levels and poised for activation [27].

Previous transcriptional profiling showed that E10.5-E11.0 XY and XX progenitor cells were nearly indistinguishable (Fig 2D), with only a handful of X- or Y-linked genes differentially expressed at this stage [13, 41]. At E10.5, we found that the vast majority of bivalent genes are shared between XY and XX progenitor cells (3545/3788 (XY) and 3545/4561 (XX)) (S2C Fig). The larger number of ovary-specific bivalent genes is most likely due to the larger number of sequencing reads obtained from the XX progenitor samples.

By E13.5, differentiation into either Sertoli or pregranulosa cells is accompanied by the upregulation of a number of Sertoli-promoting or pregranulosa-promoting genes (Fig 2G). Consistent with this, the number of bivalent promoters decreased from E10.5 to E13.5 in both XX and XY cells, whereas the number of H3K4me3-only and H3K27me3-only promoters increased (Fig 2F and 2G, and S2A Fig). Comparison of XX and XY samples showed that there is a reduction in the number of overlapping bivalent promoters between XY and XX cells, from 3545 genes at E10.5 to 2963 genes at E13.5 (S2D Fig). These data suggest that different bivalent genes resolve in Sertoli and pregranulosa cells and promote divergence during differentiation.

### Key sex-determining genes are poised at the bipotential stage

**Fig 3:**
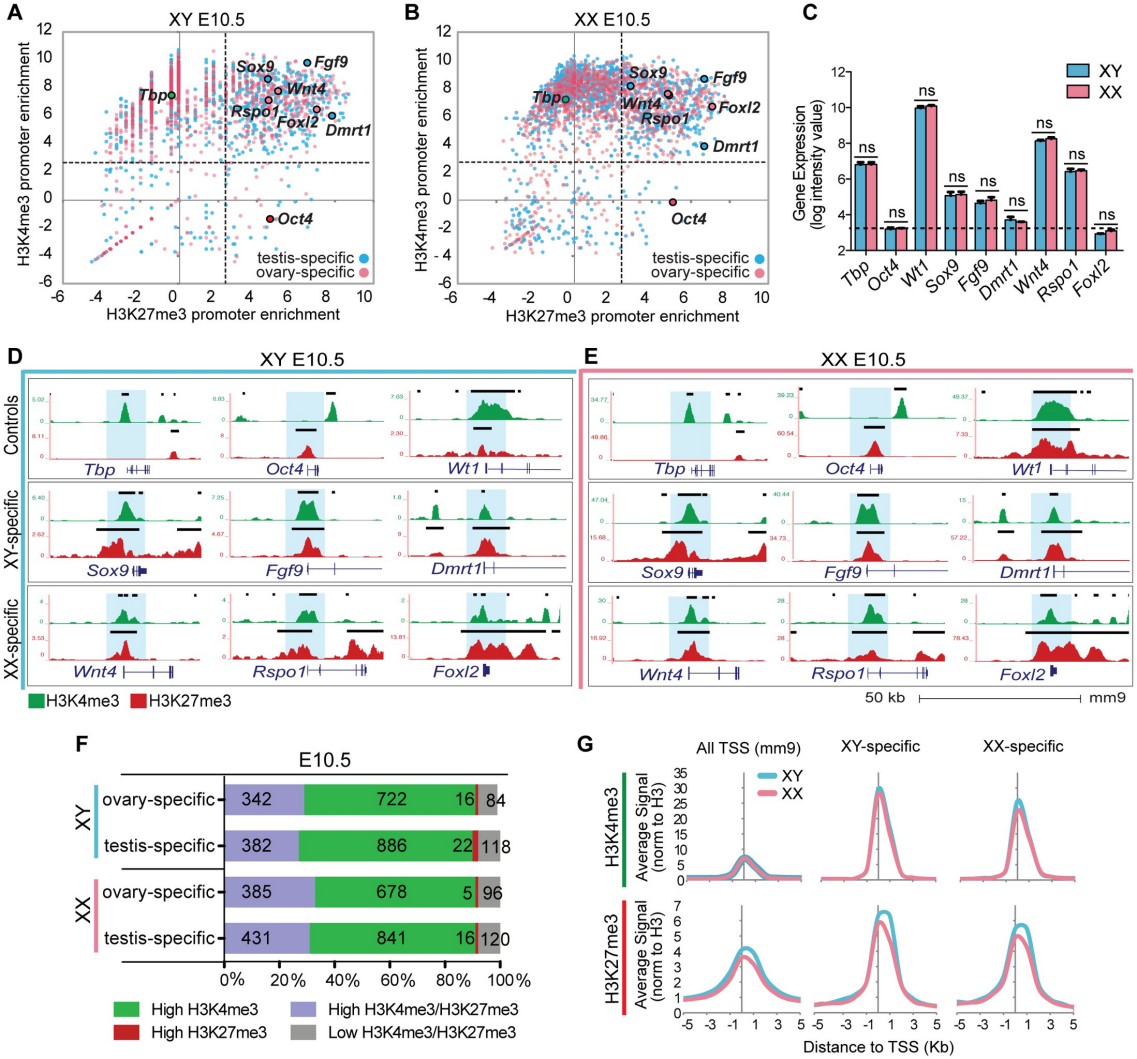
Key SD promoters are marked by both high H3K27me3 and H3K4me3 levels of enrichment prior to sex determination. (A&B) Scatterplots depicting enrichment of H3K27me3 (x axis) and H3K4me3 (y axis) normalized to total H3 within a 4kb promoter region around all TSS (mm9), in XY (left) and XX (right) progenitor cells. Genes that become either testis- or ovary-specific at E13.5 (Jameson et al., 2012b) are blue or pink respectively. *Oct4* is a germ-cell specific gene that is not expressed in supporting cells; *Tbp* is a constitutively active gene. Note that promoters of many sex determination genes (*Sox9*, *Fgf9*, *Dmrt1*, *Wnt4*, *Rspo1*, *Foxl2*) have high (log2>2.5) H3K27me3 and H3K4me3 enrichment. (C) Bar graphs denoting gene expression log intensity values from [41] for select genes in XY (blue) and XX (pink) progenitor cells. Differences between XY and XX for each gene are not significant as determined by student’s t test. Values represent mean ± SEM. (D&E) Genome browser tracks showing ChIP-seq profiles for H3K4me3 (green) and H3K27me3 (red) in XY (blue, left) and XX (pink, right) progenitor cells. Bold black lines represent significant enrichment when compared to flanking regions as determined by HOMER. Note that SD gene promoters are marked by both histone modifications. (F) Percentage of total promoters marked by high enrichment levels (>2.5 log2 enrichment normalized to H3) of H3K27me3 (red), H3K4me3 (green), both (purple), or neither (grey) throughout sex determination. Numbers of genes in each category are shown (1164 total ovary-specific and 1408 total testis-specific genes). (G) Average H3K4me3 (top) and H3K27me3 (bottom) signal normalized to H3 around the TSS (5kb upstream to 5kb downstream) (x axis) at all TSS (mm9), testis-specific promoters, and ovary-specific promoters, in XY (blue) and XX (pink) progenitor cells.

Our results revealed that 15–17% of transcription start sites (TSS) in both XX and XY supporting cells were co-marked by H3K27me3 and H3K4me3 during the bipotential stage of gonad development (S2B Fig). Bivalent genes play a crucial role in maintaining pluripotency by fine-tuning the timing of gene expression and ensuring the correct lineage commitment [28]. Despite extensive transcriptional profiling of progenitor cells [13, 41], the mechanisms that establish and maintain their bipotential state for ~24hrs are not well understood. We hypothesized that key sex-determining genes that drive either Sertoli or pregranulosa differentiation are preferentially in the bivalent category at E10.5, held in a poised state for either activation or repression following expression (or absence) of *Sry*. To investigate this hypothesis, we used the set of genes that become either Sertoli- or pregranulosa-specific at E13.5 [14], surveyed H3K4me3 and H3K27me3 deposition at E10.5, and compared the patterns to control genes whose expression is not specifically associated with the Sertoli or pregranulosa pathway (Fig 3A and 3B). Promoters were differentially enriched for H3K4me3 and H3K27me3 consistent with their expression patterns. For example, the promoter of the constitutively expressed *TATA-Box Binding Protein (Tbp)* was marked by high H3K4me3 and lacked H3K27me3 in both XY and XX supporting cells (Fig 3A–3E). In contrast, the promoter of the germ-cell specific gene *Oct4*, which is repressed in the supporting cell lineage, is marked by high H3K27me3 and lacks H3K4me3 (Fig 3A–3E). Less than 5% of both Sertoli- and pregranulosa-specific gene promoters were marked by H3K27me3-only (Fig 3F). In fact, the vast majority harbored some degree of both H3K4me3 and H3K27me3 enrichment, and approximately 30% of Sertoli- and pregranulosa-specific genes had “high” enrichment (>2.5 log2 enrichment normalized to total H3) of both histone modification marks (Fig 3A, 3B and 3F), suggesting a role for bivalent promoters in maintaining the plasticity of supporting progenitor cells to follow either of two fates.

In accordance with our hypothesis, promoters of key testis-determining genes such as *Sox9*, *Fgf9* and *Dmrt1*, and key ovary-determining genes *Wnt4*, *Rspo1* and *Foxl2* were bivalent prior to sex determination in both sexes (Fig 3A–3E). Genes such as *Wt1*, which are crucial for gonadal development in both sexe*s*, were also bivalent at E10.5 (Fig 3D and 3E). The observed bivalency in both sexes is consistent with the similar levels of expression between XY and XX progenitor cells (Fig 3C). The enrichment level of H3K27me3 and H3K4me3 modifications of bivalent genes very closely mirrors their gene expression levels. For example, *Foxl2* and *Dmrt1* are repressed at this stage with levels similar to the negative control *Oct4* (Fig 3C), and have a higher H3K27me3/H3K4me3 ratio than other sex-determining genes (Fig 3A and 3B). In contrast, genes such as *Sox9*, *Fgf9*, *Wnt4* and *Rspo1* have higher levels of expression (Fig 3C) and, accordingly, a higher H3K4me3/H3K27me3 ratio (Fig 3A and 3B). However, despite the differences in expression levels, sex determination genes have high enrichment levels of both H3K27me3 and H3K4me3, suggesting that there is transcriptional variability amongst bivalent genes as has been previously reported [27].

With the exception of a few genes linked to the sex chromosomes [13, 14], XY and XX progenitor cells are transcriptionally indistinguishable at E10.5-E11.0 [41]. At this stage, genes that later play a crucial role during sex determination are expressed at similar levels (Fig 3C). Interestingly, in a population of bipotential cells of the ventral foregut endoderm cells that give rise to either liver or pancreatic cell types, certain histone modifications can predetermine cell fate [42]. We therefore asked whether gonadal progenitor cells are epigenetically predisposed to their testis or ovary fate. However, no significant differences were observed between XY and XX progenitor cells for mean enrichment levels of H3K4me3 and H3K27me3 at sex-determining genes (Fig 3G), suggesting that promoter deposition of H3K4me3 and H3K27me3 does not direct differentiation of XY and XX progenitor cells, but rather contributes to their bipotential state.

Our results suggest that although only 17–20% of all annotated genes are bivalent at E10.5 (S2A Fig), ~30% of genes that later become Sertoli-specific as well as ~30% of genes that later become pregranulosa-specific are bivalent in both XX and XY progenitor cells, suggesting that bivalency plays a large role in the regulation of sex-determining genes.

### Repressed key sex-determining genes remain poised after sex determination

**Fig 4:**
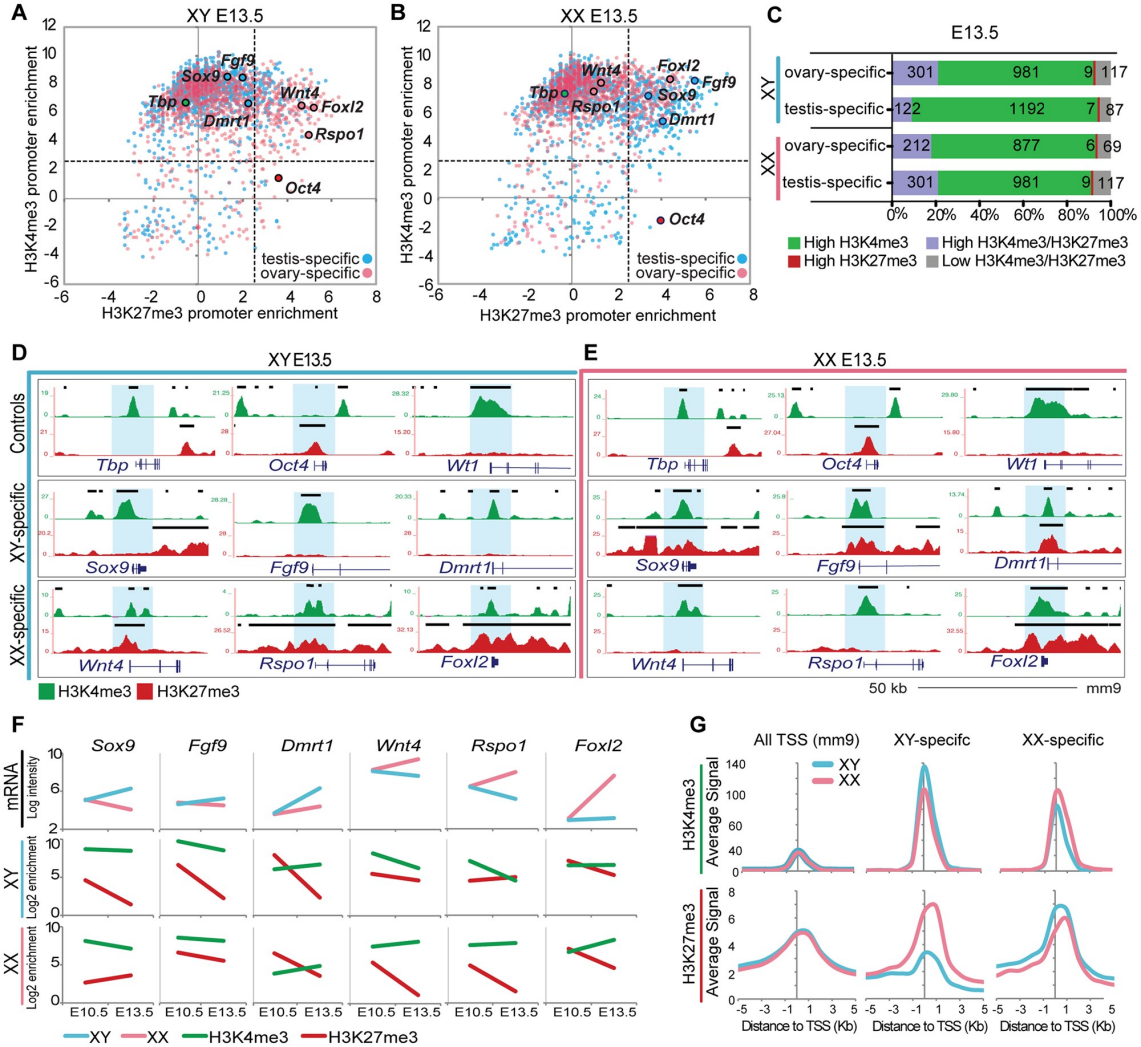
Repressed key SD promoters retain both high H3K27me3 and H3K4me3 levels of enrichment after sex determination. (A&B) Scatterplots depicting enrichment of H3K27me3 (x axis) and H3K4me3 (y axis) normalized to total H3 at a 4kb promoter region, in Sertoli cells (left) and pregranulosa cells (right) at E13.5. Genes that become either testis or ovary-specific at E13.5 as determined by [14] are in blue and pink respectively. Note that promoters of SD genes that promote the alternate fate have high (log2>2.5) H3K27me3 and H3K4me3 enrichment even after sex determination. (C) Percentage of total promoters marked by high enrichment levels (>2.5 log2 enrichment normalized to H3) of H3K27me3 (red), H3K4me3 (green), both (purple), or neither (grey) throughout sex determination. Numbers of genes in each category are shown (1164 total ovary-specific and 1408 total testis-specific genes). (D&E) Genome browser tracks showing ChIP-seq profiles for H3K4me3 (green) and H3K27me3 (red) in Sertoli cells (blue, left) and pregranulosa cells (pink, right) at E13.5. Bold black lines represent significant enrichment when compared to flanking regions as determined by HOMER. Note that repressed key SD genes are marked by both histone modifications. A closer view of *Foxl2* is in S3B Fig. (F) Time course of gene expression (log intensity values from [41]) from E10.5 to E13.5 in XY (blue) and XX (pink) supporting cells (top row), and of H3K4me3 (green) and H3K27me3 (red) promoter enrichment value normalized to H3 in XY (middle row) and XX (bottom row) supporting cells. (G). Average H3K4me3 (top three) and H3K27me3 (bottom three) signal normalized to H3 around the TSS (5kb upstream to 5kb downstream) (x axis) at all TSS (mm9), testis-specific promoters, and ovary-specific promoters in Sertoli (blue) and pregranulosa (pink) cells.

Having established that key sex-determining genes are bivalent prior to sex determination, we next asked whether their promoters resolve into sex-specific patterns of H3K4me3 and H3K27me3 after sex determination, consistent with the upregulation of either the testis or ovary pathway and repression of the alternate fate. To investigate this, we compared the genome-wide profile of H3K4me3 and H3K27me3 in purified Sertoli and pregranulosa cells from E13.5 XY and XX gonads. We show that upregulation of sex-determining genes is accompanied by a strong reduction of the repressive H3K27me3 mark at their promoters by E13.5 (Fig 4A–4F). For example, the key testis-determining genes *Sox9*, *Fgf9* and *Dmrt1*, which become upregulated in E13.5 XY supporting cells following expression of *Sry* (S3A Fig), show a >2-fold reduction of H3K27me3 enrichment in Sertoli cells (Fig 4F). In total, 260 of the 382 (68%) testis-determining genes that were bivalent at E10.5 lose H3K27me3 enrichment and shift towards an H3K4me3-only state in Sertoli cells, consistent with an upregulation of Sertoli-specific gene transcription (Fig 4A and 4C). Conversely, the key ovary-determining genes *Wnt4*, *Rspo1* and *Foxl2*, which become upregulated in E13.5 XX supporting cells in the absence of *Sry* (S3A Fig), show a >2-fold reduction of H3K27me3 enrichment in pregranulosa cells (Fig 4F). Accordingly, 173 of the 385 (45%) ovary-determining genes that were bivalent at E10.5 shift towards an H3K4me3-only state in pregranulosa cells by E13.5 (Fig 4B and 4C). Promoter H3K4me3 enrichment at these genes does not significantly increase during sex determination, but rather is retained at similar levels (Fig 4F). It is important to note that *Foxl2*, a small single-exon gene, is embedded within an H3K27me3-dense locus and appears to retain H3K27me3 in pregranulosa cells. However, a closer look at the TSS and gene body itself reveals that these regions do in fact lose H3K27me3 upon differentiation (S3B Fig), although this is not evident in our scatterplot analyses that used a 4kb window around the TSS for calculating enrichment levels.

Remarkably, we found that sex-determining genes that promote the alternate cell fate and become transcriptionally repressed during sex determination did not lose their active H3K4me3 mark and resolve into H3K27me3-only promoters. Instead, repressed sex-determining genes remained bivalent even after progenitor cells had departed from their bipotential state and differentiated into either Sertoli or pregranulosa cells. For example, *Wnt4*, *Rspo1* and *Foxl2*, ovary-determining genes that are actively repressed in Sertoli cells (S3A Fig), retain high enrichment of both H3K4me3 and H3K27me3 (Fig 4A and 4D). Similarly, *Sox9*, *Fgf9*, and *Dmrt1*, testis-determining genes that are not upregulated in pregranulosa cells (S3A Fig), retain high enrichment of both H3K4me3 and H3K27me3 (Fig 4B and 4E). To determine whether this bivalency at repressed genes is retained in adult stages, we performed ChIP-qPCR in purified adult Sertoli cells (>2 m/o males) and found that the ovary-specific genes *Wnt4*, *Rspo1*, *Foxl2*, *Bmp2* and *Fst* still retained enrichment of both H3K27me3 and H3K4me3 (S4A Fig). Given the larger amount of available material, bivalency was further validated by ChIP-re-ChIP in adult testes (S4B Fig).

These results are not exclusive to these key sex-determining genes. In fact, 242 of the 342 (71%) bivalent ovary-determining genes at E10.5 remain bivalent at E13.5 in Sertoli cells (Figs 3F, 4A and 4C), and 301 of the 431 (70%) of bivalent testis-determining genes at E10.5 remain bivalent at E13.5 in pregranulosa cells (Figs 3F, 4B and 4C). These results are reflected in the analyses of the mean enrichment level of H3K4me3 and H3K27me3 at the promoters of testis- and ovary-specific genes in Sertoli and pregranulosa cells (Fig 4G). While average H3K4me3 enrichment is higher at testis-specific genes in Sertoli cells and at ovary-specific genes in pregranulosa cells, genes promoting the alternate cell fate have not completely lost H3K4me3, suggesting that even repressed genes retain this active mark. However, the average H3K27me3 enrichment level is higher at ovary-specific genes in Sertoli cells and at testis-specific genes in pregranulosa cells, consistent with its role in maintenance of the repressed state (Fig 4G). Interestingly, there is a significant difference between the mean H3K27me3 enrichment at ovary-determining genes in Sertoli cells and pregranulosa cells, whereas this difference is not as marked at testis-determining genes. This may be a reflection of the total percentage of bivalent genes that have not resolved at E13.5 (Figs 3F and 4C): while 260 of the initial 382 (68%) of testis-specific bivalent genes have resolved in E13.5 Sertoli cells, only 219 of the initial 431 (51%) of ovary-specific bivalent genes have resolved in E13.5 pregranulosa cells, possibly because the ovary developmental pathway is not yet fully established at E13.5 [14]. Interestingly, although levels of H3K27me3 remain constant at genes that retain bivalency from E10.5 to E13.5, the transcription levels of some of these genes decrease (e.g. *Wnt4* and *Rspo1* in Sertoli cells in Fig 4), pointing towards a possible secondary silencing mechanism.

Our results suggest that key sex-determining genes that promote the alternate cell fate remain in a poised state for activation even after sex determination has occurred, possibly providing supporting cells with an epigenetic memory of their bipotential state and contributing to their ability to transdifferentiate long after sex determination [15, 16]. Furthermore, the finding that H3K27me3 does not change at ovary-promoting genes during sex determination suggests that stabilization of the testis fate requires maintenance of existing H3K27me3 marks rather than establishment of these marks after commitment to the testis fate.

### Sex reversal is rescued in *Cbx2;Wnt4* double knock-out mice

Our results show that H3K27me3 increases or is stably maintained at promoters of repressed genes during sex determination with evidence for expansion across loci, a known role of the PcG complex (S5 Fig) [43]. Investigation of pregranulosa-promoting genes in Sertoli cells revealed that genes with known roles in ovary development, such as *Foxl2*, *Lhx9*, *Irx3*, *Lef1*, *Bmp2*, *Rspo1*, *Wnt2b* and *Wnt4*, are amongst the genes with the highest levels of H3K27me3 enrichment (S6 Fig), suggesting that stable repression of these genes is critical in Sertoli cells. Functional annotation of H3K27me3+ pregranulosa genes revealed that the Wnt signaling pathway is highly represented (S7A Fig) and confirmed that the main targeted biological process is the female reproductive system (S7B Fig). In fact, in addition to *Wnt4* and *Rspo1* (Fig 4), several other members of the Wnt signaling pathway that become ovary-specific, such as *Wnt2b*, *Axin2*, *Lef1*, and *Lgr5* [14], are bivalent with high levels of H3K27me3 at their promoters (S7C Fig). As *Wnt4* is a known ovary-promoting/anti-testis gene [12], our results suggest that the XY sex reversal observed following loss of *Cbx2* could be due to a failure to repress the Wnt signaling pathway in Sertoli cells.

**Fig 5:**
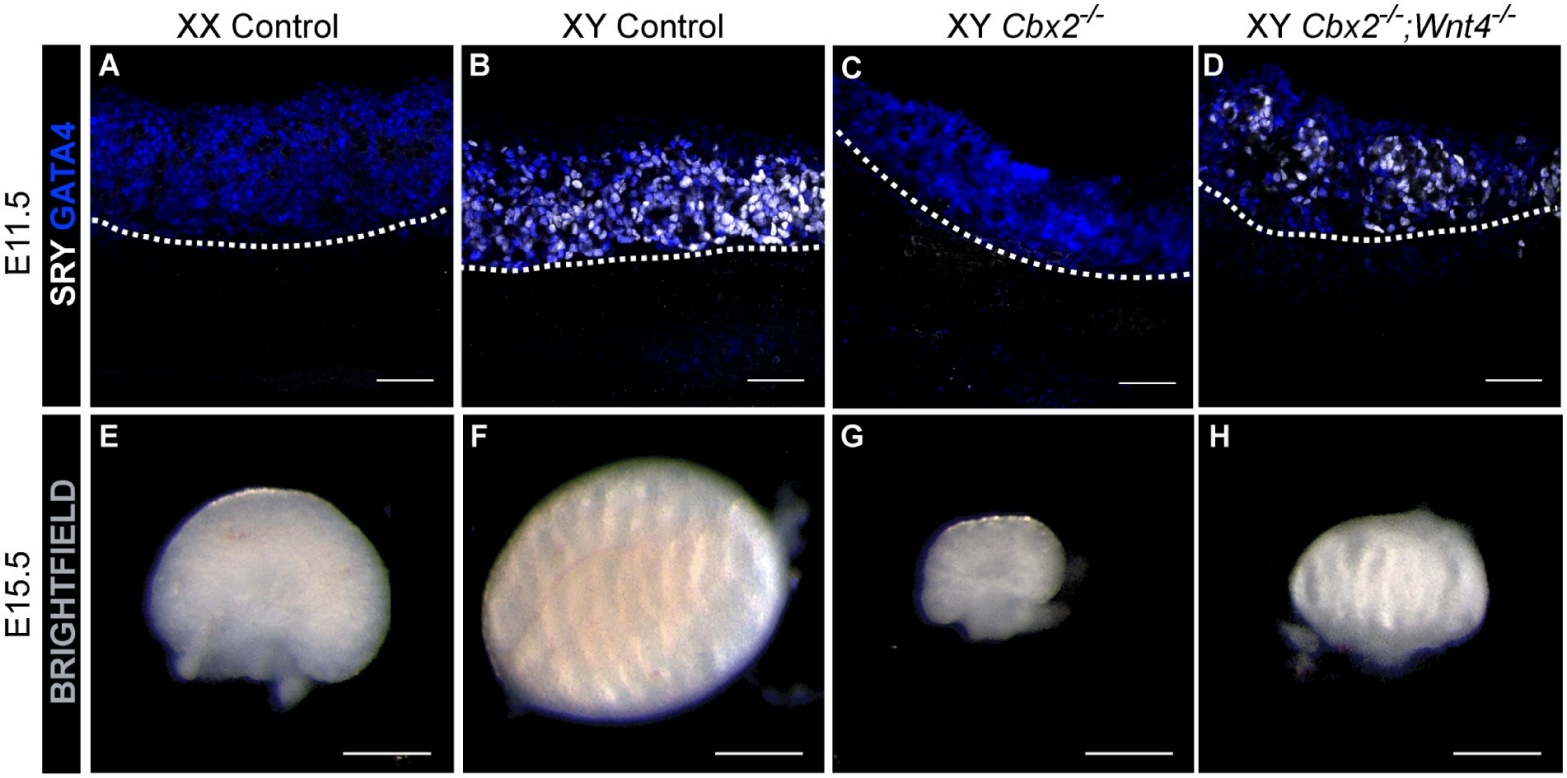
*Sry* expression and testis development are rescued in XY gonads of *Cbx2;Wnt4* double knockout (dKO) mice. Immunofluorescent analysis of gonads from E11.5 (A-D) and brightfield images of E15.5 (E-H) gonads. E11.5 gonads (A-D) are stained with somatic cell marker GATA4 (blue) and SRY (white). Loss of *Cbx2* in E11.5 XY gonads leads to loss of SRY (C). SRY is rescued in *Cbx2;Wnt4* dKO gonads (D). Bright field images of E15.5 gonads show the morphology of WT ovaries (E) and WT testis (F). Loss of *Cbx2* causes XY sex reversal leading to formation of functional but hypoplastic ovaries (G). Loss of *Wnt4* on a *Cbx2* KO background recues testis morphology, but not testis size (H). Images correspond to one of n = 3. Scale bars represent 50μm (A-D) and 500μm (E-H).

To test whether loss of *Wnt4* could rescue testis development in the *Cbx2*^*-/-*^ XY gonad, we created a *Cbx2*^*-/-*^*;Wnt4*^*-/-*^ double knockout mouse. In accord with our hypothesis, immunofluorescent analysis of E11.5 XY *Cbx2*^*-/-*^*;Wnt4*^*-/-*^ gonads showed SRY expression (Fig 5D), similar to wild-type XY mice (Fig 5B). Furthermore, there was an increase in SOX9-positive cells, and testis cord formation was rescued in E13.5 *Cbx2*^*-/-*^*;Wnt4*^*-/-*^ gonads (S8 Fig), which develop as testes (Fig 5H and S8D Fig). These results suggest that, at least in the absence of *Wnt4*, *Cbx2* is not required for *SRY* activation as previously proposed. Instead, a failure to repress the ovary pathway as Sertoli cells differentiate has the indirect effect of blocking the stabilization of *Sry* and *Sox9* expression.

It was originally reported that *Cbx2*^*-/-*^ gonads were hypoplastic due to a somatic cell proliferation defect [33]. Despite the fact that XY *Cbx2*^*-/-*^*;Wnt4*^*-/-*^ gonads express testis markers and are morphologically similar to XY wild type gonads (Fig 4D and 4H), gonad size is not rescued, consistent with the idea that *Cbx2* regulates sex determination and gonad size through independent pathways [33]. Our results suggest that CBX2 positively regulates *Sry* and *Sox9* expression by directly repressing the pregranulosa-promoting Wnt signaling pathway.

### CBX2 targets the downstream Wnt signaler *Lef1* for repression

**Fig 6:**
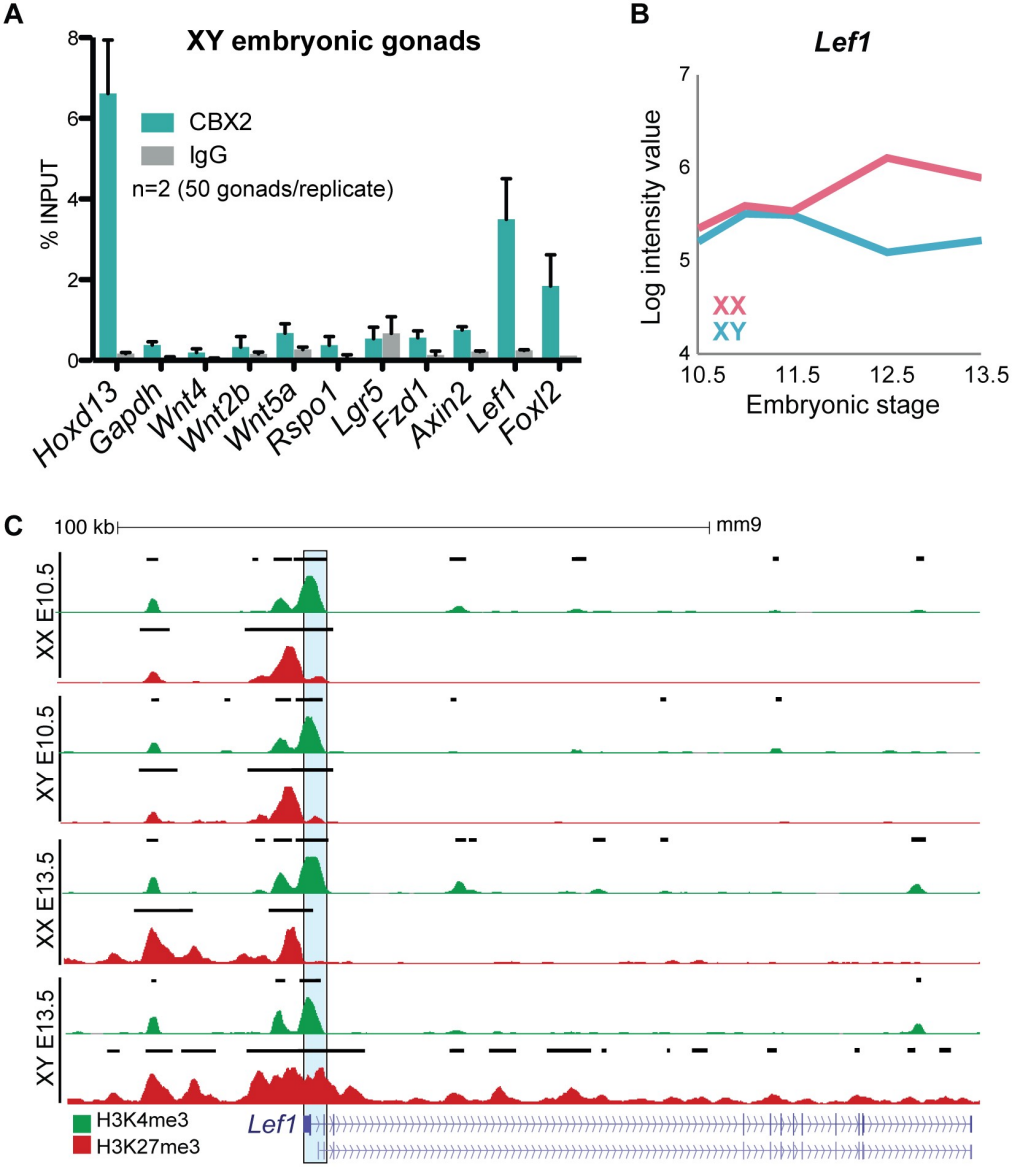
CBX2 targets *Lef1* for repression in XY gonads. (A) ChIP-qPCR for CBX2 on embryonic gonads (2 replicates containing 50 pooled E13.5 and E14.5 XY gonads). Values represent mean ± SEM. (B) Gene expression profile of *Lef1* in XX (pink) and XY (blue) supporting cells throughout sex determination from [41]. (C) Genome browser tracks showing ChIP-seq profiles for H3K4me3 (green) and H3K27me3 (red) at the *Lef1* locus. Note the spreading of H3K27me3 over the gene body in XY E13.5. Bold black lines represent significant enrichment when compared to flanking regions as determined by HOMER.

To determine whether CBX2 directly targets *Wnt4* and/or other Wnt signalers, we performed ChIP-qPCR in pooled E13.5 and E14.5 XY gonads (Fig 6A), and in adult (>2m/o) testes (S9 Fig). *Hoxd13*, a known CBX2 target gene [23], was used as a positive control. In contrast, the promoter region of the constitutively active gene *Gapdh* was used as a negative control. Surprisingly, CBX2 did not bind *Wnt4*, or other Wnt signalers such as *Wnt2b*, *Wnt5a*, *Rspo1*, *Axin2*, *Fzd1*, and *Lgr5*. However, *Lef1* was significantly targeted by CBX2 in both embryonic and adult testes. Although CBX2 is expressed in all gonadal cell types throughout sex determination, gene expression analyses performed in several cell lineages of the gonad show that *Lef1* is repressed specifically in the male supporting lineage [14], suggesting that the enrichment of CBX2 at the promoter of *Lef1* in whole testes is likely coming from Sertoli cells. Downstream of Wnt, LEF1 interacts with β-catenin in the nucleus to drive expression of target genes [44]. During sex determination, *Lef1* is silenced in Sertoli cells and becomes ovary-specific (Fig 6B) [14, 41]. Accordingly, as *Lef1* is silenced in Sertoli cells, H3K27me3 spreads upstream and downstream off the TSS as well as over the gene body. In pregranulosa cells, H3K27me3 is removed from the TSS (Fig 6C). Importantly, despite increased levels of H3K27me3 in Sertoli cells, *Lef1* retains H3K4me3 enrichment at its promoter from the progenitor state (Fig 6C). Our results suggest that CBX2 inhibits upregulation of the ovary pathway during testis development, and possibly maintains Sertoli cell identity in adulthood, at least in part by stably repressing WNT4’s downstream target *Lef1*.

## Discussion

The early progenitors in the bipotential mouse gonad are a valuable model for the investigation of how cells resolve their fate and commit to one of two differentiation pathways. We show that XY cells that lack CBX2, the cPRC1 subunit that binds H3K27me3 and mediates chromatin compaction, fail to repress the ovary pathway, suggesting that cPRC1 is critical for regulating the testis vs. ovary cell-fate decision of the fetal gonad. To provide insight into the epigenetic mechanisms that regulate this cell-fate decision, we developed an *in vivo* quantitative profile of H3K4me3 and H3K27me3 enrichment in XY and XX gonadal supporting cells at time points before (E10.5) and after (E13.5) sex determination. We show that genes essential to establish both testis and ovary fate are initially poised in both the XX and XY gonad. Once sex determination occurs, the testis- or ovary-expressed bivalent genes resolve into H3K4me3-only promoters. However, the genes associated with the alternate sexual fate retain their bivalent marks, supporting the importance of a stable repressive mechanism that prevents their upregulation, and ensures that the correct lineage is followed during sex determination. We show that XY sex-reversal caused by loss of CBX2 can be rescued by simultaneous loss of *Wnt4*, and that CBX2 directly binds *Wnt4’s* downstream target *Lef1*, an ovary-specific co-factor of β-catenin that remains bivalent in committed Sertoli cells. These findings suggest that *Cbx2* is required during sex determination to stabilize the testis fate by blocking the upregulation of bivalent ovary pathway genes.

**Fig 7:**
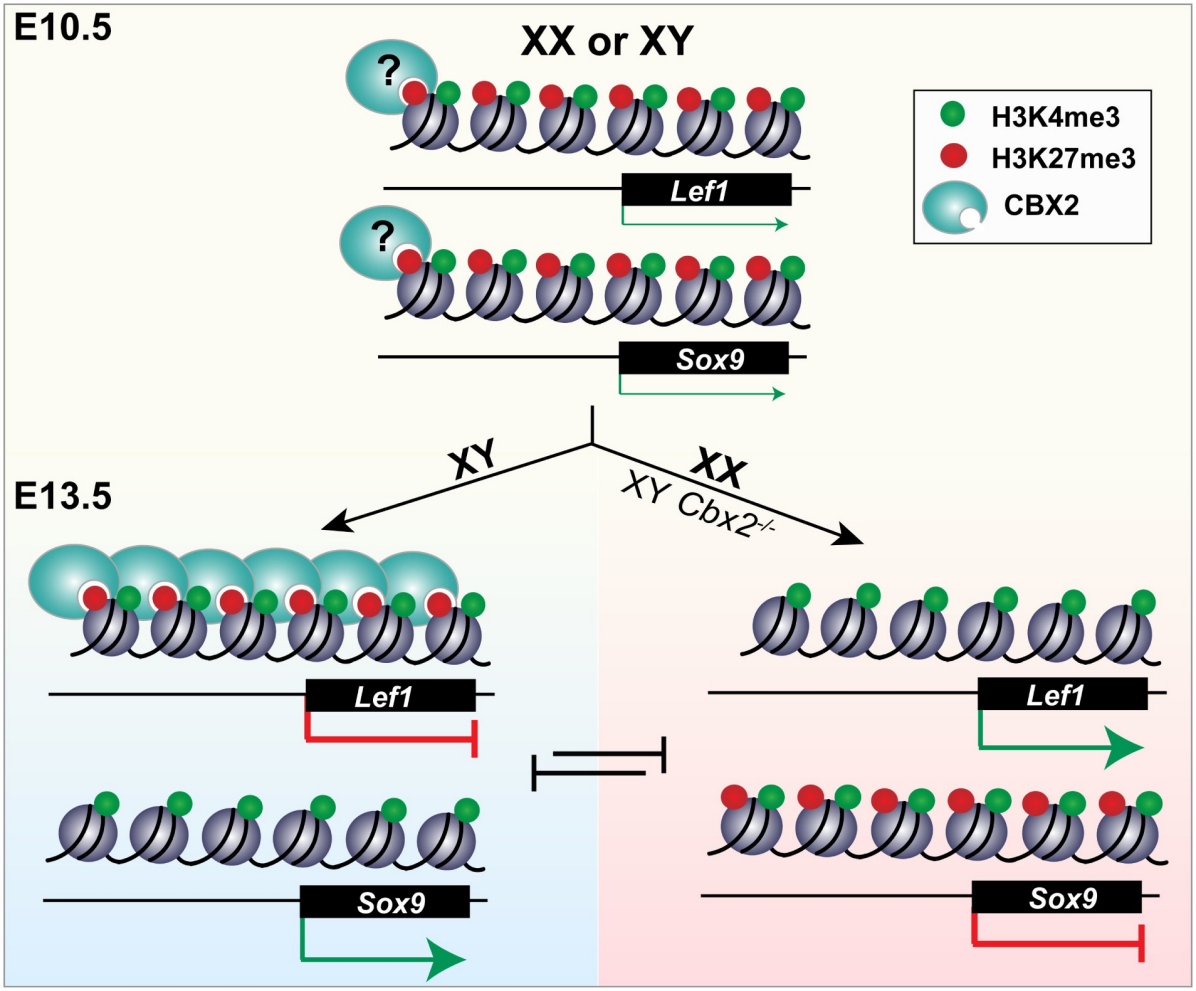
Model of the epigenetic regulation of mammalian sex determination. At the bipotential stage (E10.5), testis- (eg. *Sox9)* and ovary-determining (eg. *Lef1*) genes are bivalent, marked by both H3K27me3 and H3K4me3. Bivalent SD genes are co-expressed at low levels, poised for expression of *Sry* and commitment to the testis fate (XY, blue) or in absence of *Sry*, commitment of the ovary fate through the Wnt signaling pathway (XX, pink). Upregulation of SD genes is accompanied by loss of H3K27me3. Genes that promote the alternate fate and are repressed after sex determination (E13.5) remain bivalent. CBX2 binds to Wnt’s downstream target *Lef1* in XY gonads, inhibiting its upregulation and stabilizing the testis fate. In XX E13.5 gonads, or in XY gonads that lack *Cbx2*, *Lef1* promotes pregranulosa development which blocks upregulation of the testis fate (right, pink). It remains unclear whether CBX2 maintains H3K27me3 from the progenitor state in XY cells and is removed from specific targets in XX cells, or whether it is targeted specifically to ovary genes during Sertoli cell development.

The co-occurrence of the active H3K4me3 and the repressive H3K27me3 marks was first reported in ESCs, in which a cohort of developmental genes is “bivalent”, poised for future repression or activation [27–29]. Our findings are consistent with the idea that epigenetic regulation plays a critical role in maintaining pluripotency and facilitating divergence into multiple differentiated states. We show that most genes that become either testis- or ovary-specific after sex determination initially harbor both the H3K4me3 and H3K27me3 modifications at their promoters. More specifically, key sex-determining genes (genes known to cause sex disorders when disrupted) such as the testis-determining genes *Sox9*, *Fgf9* and *Dmrt1* and the ovary-determining genes *Wnt4*, *Rspo1* and *Foxl2* have high enrichment levels of both chromatin marks at the bipotential stage (>log 2.5 enrichment normalized to total H3). Our findings suggest that sex-determining genes that are initially repressed or lowly expressed at similar levels between XY and XX gonads are bivalent in progenitor cells, held in a chromatin-accessible conformation (Fig 7). This state maintains the balance between the testis and ovary fate yet renders genes responsive to the sex-determining signal triggered by *Sry* in XY gonads or *Wnt4/Rspo1* in XX gonads.

Typically, upregulation of bivalent genes is accompanied by loss of H3K27me3 at the promoter, whereas repression is accompanied by a loss of H3K4me3 [27]. This binary resolution maintains the transcriptional state throughout cell divisions even in absence of the initial signal. We therefore predicted that sex-determining genes would resolve into sex-specific patterns of histone modifications, stably transmitting the cell fate decision throughout adulthood. Instead, we show that although sex-determining genes that become upregulated do lose their repressive mark, sex-determining genes that promote the alternate pathway and become transcriptionally repressed persist in a poised state even after differentiation into either Sertoli or pregranulosa cells (Fig 7). The finding that ovary-determining genes retain enrichment of both H3K4me3 and H3K27me3 in Sertoli cells purified from adult testes led us to speculate that transmission of poised sex-determining genes from the bipotential to the differentiated state could confer the remarkable plasticity that enables *in vivo* transdifferentiation of the supporting cell lineage to the alternate cell fate, even long after the fate-decision has been made [15, 16]. Our findings may also explain why repression of the alternate cell fate is an active process throughout adulthood. In the absence of active repressors, poised promoters may be vulnerable to differentiation toward the alternate cell fate. Our data suggest that maintenance of a bivalent but silent state (i.e. maintenance of H3K27me3) requires the activity of cPRC1.

Accordingly, loss of the cPRC1 subunit CBX2 leads to upregulation of the ovary pathway and complete XY sex reversal. This was originally thought to be due to a failure to activate *Sry* [33]. Although some PcG proteins have been reported to promote activation of certain targets [45–47], our observation that XY *Cbx2*^*-/-*^ cells co-expressed Sertoli and pregranulosa markers led us to hypothesize that downregulation of the testis pathway is the indirect consequence of failure to repress the ovarian program. Our present results are consistent with this hypothesis. Although we did not capture SRY expression in XY *Cbx2* single mutant samples, the most parsimonious explanation for the presence of SOX9 is that *Sry* was transiently expressed, possibly with a delay or at lower levels. In addition, we do show that SRY is present in the absence of *Cbx2* in *Cbx2;Wnt4* double mutant gonads. These data suggest that *Cbx2* is not required for *Sry* activation as was previously proposed, or that this role is redundant in the absence of canonical WNT signaling. These results point towards CBX2 as a stabilizer of *Sry* and the testis pathway. Several lines of study have shown that the bipotential gonad is primed towards the female pathway, suggesting that the gonad is on an ovary trajectory before *Sry* is activated [13, 14, 48]. The failure to establish the testis pathway if expression of *Sry* is delayed suggests that the opportunity to divert the gonad to testis fate is strictly time-limited [49]. In addition to the time-constraint, levels of *Sry* must reach a critical threshold required to establish Sertoli cell differentiation [50]. Although it is not yet clear what establishes these limits, one hypothesis is that it is the escalation of the female pathway possibly driven by Wnt signaling. We speculate that the role of bivalency is to maintain testis- and ovary-determining genes in balance, repressed but poised for activation upon receipt of a developmental signal. Therefore, we propose that in absence of CBX2, the ovary-promoting pathway is engaged early or more strongly, creating an environment in which *Sry* and the testis pathway cannot be stabilized possibly due to high nuclear levels of β-catenin. Consistent with this interpretation, we show that CBX2 represses *Wnt4’s* downstream target *Lef1* (Fig 7). While it would be surprising if *Lef1* were the sole target of CBX2 in testes, regulation of *Lef1* might be pivotal in the transition from the bipotential fate to the testis pathway. Evidence for this comes from the finding that SOX9 and Tcf-Lef compete for binding sites within β-catenin: whereas the interaction between SOX9 and β-catenin induces β-catenin degradation, binding between Tcf-Lef and β-catenin leads to the upregulation of Wnt target genes [51, 52]. Although levels of LEF1 may be important for tipping the balance of the bipotential gonad towards the ovary fate, other targets of CBX2 may also be important.

A smaller, but still significant number of genes associated with testis development are repressed in XX cells, thus it is still unclear why loss of *Cbx2* does not disrupt ovary development despite its similar expression levels in XY and XX supporting cells [14]. One possibility is that wide-spread epigenetic mechanisms have evolved in XY gonads to repress the ovary pathway, consistent with the fact that more pregranulosa-promoting genes are expressed at the bipotential stage than Sertoli-promoting genes, and that repression of pregranulosa genes is a more significant component of the testis pathway [13, 14]. Alternatively, it is possible that maintenance of H3K27me3 at Sertoli-specific genes in XX cells is mediated by CBX subunits other than CBX2, as CBX4, CBX6 and CBX8 are expressed at similar levels between Sertoli and pregranulosa cells throughout sex determination [14, 41]. This may also be the case for H3K27me3-positive Wnt pathway genes that we found are not bound by CBX2 in our CBX2 ChIP-qPCR. Further insight into the mechanistic functions of CBX2, such as whether cPRC1 achieves specificity by interacting with sex-specific transcription factors or non-coding RNAs during sex determination, will benefit from the integration of various genome-wide binding profiles of histone modifications and sex-determining transcription factors that are emerging in the field [53, 54]. Furthermore, identification of additional CBX2 targets by ChIP-seq will benefit from more advanced techniques compatible with small amounts of starting material.

Our results highlight the importance of epigenetic mechanisms in the establishment and resolution of the bipotential state of gonadal precursors. With the development of an *in vivo* genome-wide epigenetic profile of the supporting cell lineage throughout sex determination, we provide insight into how the bipotential state is established, and an explanation for how differentiated supporting cells retain an epigenetic memory of their bipotential state. Furthermore, we describe a widespread role for the PcG proteins in repressing the ovary pathway during testis development and show that its subunit CBX2 is required to directly repress *Wnt4’s* downstream target *Lef1* in XY gonads. Expanding our knowledge of the regulatory mechanisms underlying sex determination may increase our ability to identify the cause of disorders of sex development with unknown etiologies, over 50% of which remain undiagnosed.

## Materials and methods

A complete list of primers and antibodies can be found in the supplemental materials, S1–S4 Tables.

### Mice

The *Sox9-CFP*, *TESMS-CFP* and *Sf1-eGFP* reporter mouse lines, and the *Cbx2*, *Fgf9* and *Wnt4* KO mouse lines were previously generated [35, 39–41, 55] and maintained on a C57BL/6 (B6) background. Timed matings were established between genotypes and CD1 females. The morning of a vaginal plug was considered E0.5. Embryos were collected at E10.5-E13.5 and genotyped by PCR for mutant alleles. Genetic sex was determined using PCR for the presence/absence of a region of the Y chromosome (see primer list for details).

### Immunofluorescence

Embryonic gonads were dissected and fixed for 1hr at RT in 4% paraformaldehyde (PFA). Whole gonads were stained as previously described [56] using the following primary antibodies: SRY (1:100, gift of Dagmar Wilhelm, The University of Melbourne, Melbourne, Australia), SOX9 (1:3000, EMD Millipore, Damstadt, Germany), FOXL2 (1:250; Novus Biologicals, Littleton, CO), GATA4 (1:100, Santa Cruz Biotechnology, Santa Cruz, CA), PECAM1 (1:250; BD BioSciences, San Jose, CA). Alexa Fluor488-labeled anti-rat or anti-mouse (Life Technologies, Carlsbad, CA), Alexa Fluor647-labeled anti-goat (Life Technologies, Carlsbad, CA) and Cy3-labeled anti-rabbit (Jackson ImmunoResearch Laboratories, Inc., West Grove, PA) antibodies were used as secondary antibodies (1:500). Nuclei were stained with DAPI. Whole samples were mounted in DABCO (Sigma-Aldrich) in 90% glycerol and imaged with confocal scanning microscopy using a Leica SP2.

### Adult Sertoli cell purification

Sertoli cells were isolated as in [57] using the same solutions and reagents. Briefly, whole testes were dissected from adult B6 males (>2 months old) and tunica was removed. Testis cords were gently separated and rinsed in PBS. Testis cords were then incubated in a collagenase solution to remove interstitial cells. To separate Sertoli cells from germ cells, cords were then incubated in a trypsin solution followed by an enzyme solution containing collagenase and hyaluronidase. At this point, cords were mechanically disassociated by pipetting into a single-cell suspension and washed in PBS. Cells were subjected to a hypotonic shock solution of 1:2 HBSS to H_2_O to lyse germ cells, which are smaller than Sertoli cells. Sertoli cells were recovered by centrifugation at 500rpm for 5 mins at 4°C, washed twice in PBS and resuspended in 500ul of PBS/3% BSA. Cells were then FACS-purified to collect the large cell population while eliminating contaminating germ cells, nuclei and other debris. ~400K Sertoli cells were routinely recovered from 1 adult mouse. Cells were sorted in PBS/3% BSA for native ChIP-qPCR as described below.

### Chromatin immunoprecipitation

#### ChIP-seq

XX and XY supporting cells were FACS-purified from E10.5 *Sf1-eGFP* gonads [38], E13.5 XY *SOX9-CFP* gonads [39] and E13.5 XX *TESMS-CFP* gonads [40] on the same day, and immediately processed for ChIP-seq. ChIP-seq was performed with no modifications as in Van Galen *et al*. 2016 [37] on ~30K–100K FACS-purified supporting cells from pooled gonads. 400K *Drosophila* S2 cells were added per IP as carrier chromatin. ChIP-seq was performed on two biological replicates. Chromatin was digested using 150 units of MNase (NEB #M0247S) and ChIP-seq was performed using 3μl of H3 antibody (Active Motif #39763), 3μl of H3K4me3 antibody (Active Motif #39159) or 5μl H3K27me3 antibody (CST #9733S).

#### Bioinformatics

Alignment to the mm9 mouse genome was performed using Bowtie. For visualization on the UCSC genome browser, replicates were concatenated to maximize number of reads. H3 ChIP was used as input. To identify regions significantly enriched for H3K27me3 compared to flanking regions (peaks), HOMER was used for each independent replicate using the findPeaks function and settings “—style histone”, with a size of 5000 for H3K27me3 and 1000 for H3K4me3. The setting “-C 0” was used in MNase ChIP to disable fold enrichment limit of expected unique tag positions. BigWig files were created using bedGraphToBigWig. To limit analyses only to gene promoters, Bedtools Intersect was used to intersect the ChIP-seq called peak files with a file containing all TSSs annotated in the mm9 genome downloaded from the UCSC genome browser, which was expanded to span 2kb upstream and 2kb downstream of the TSS. Tag counts for each 4kb promoter region were obtained using the HOMER annotatePeaks.pl function and were normalized to the sample’s corresponding H3 tag counts. Scatterplots of promoter tag counts normalized to H3 were generated in Excel. Histograms were generated using HOMER’s annotatePeaks.pl function with “-size 20000” and “-hist 1000”. Quantitative comparison was only performed between replicates that were processed together (i.e. between samples in replicate 1 or replicate 2).

#### Native ChIP-qPCR for histone modifications

ChIP-qPCR was modified from the ChIP-seq protocol described above using the same buffers and reagents [37]. Briefly, FACS-purified cells or 400K Drosophila S2 cells were resuspended in 20μl PBS/2xPIC (PIC, Thermo Fisher 1862209) and 20μl 2x Lysis Buffer containing 150u of Micrococcal Nuclease (NEB #M0247S). Cells were lysed on ice for 20min and chromatin was digested for 10min at 37°C. The digest was stopped by adding 40μl Dilution Buffer + EGTA to a final concentration of 25mM and incubated on ice 10min. The FACS-purified cells and the S2 cells were pooled, and the sample was brought up to 200μl/IP with Dilution Buffer/PIC. Samples were then split and incubated with 5μl of H3K4me3 antibody (Active Motif #39159), 5μl H3K27me3 antibody (CST #9733S) and 5μl IgG (CST #2729S) as a control overnight at 4°C on a tube rotator. 25μl of protG Dynabeads (Thermo Fisher 10004D) were washed 2x in Dilution Buffer/PIC, added to each IP, and incubated for an additional 3hrs at 4°C on a rotator. Samples were magnetized, and the unbound portion was recovered. Beads were washed 2x in 200μl of ice-cold RIPA, 1x in 200μl ice-cold RIPA/High Salt, 1x in 200μl ice-cold LiCl buffer and 1x in 100μl of TE by moving tubes from the front of the magnet to the back twice. Washed beads were resuspended in 100μl Elution Buffer. Beads and unbound samples were then incubated with 0.5μl of RNase Cocktail (Invitrogen AM2286) and 0.5μl of protK (10mg/ml) at 37°C for 30min and 63°C for 1 hour. DNA was purified using a MinElute Qiagen Kit (28004) and used for qPCR. qPCR was analyzed as Ratio Bound/Unbound = 1/2^(Bound CT–Unbound CT), and IgG values were subtracted. Each ChIP-qPCR was performed three times, each on cells from a pool of multiple gonads.

#### Cross-linked ChIP-qPCR for CBX2

(buffers from [37]). Whole adult testes were dissected from >2m/o mice, the tunica was removed, and tubules were dropped into a glass mortar containing 2ml of 1% PFA/PBS. The timer was set for 10min and testes cords were homogenized with a glass pestle. Homogenized tubules were transferred to a 15ml conical tube and placed on a shaker for the remainder of the 10 minutes. For embryonic gonads, gonads were removed from the mesonephros and directly incubated in 1ml of 1% PFA/PBS on a shaker for 10min at room temperature. PFA was quenched with a final concentration of 125mM glycine and incubated an additional 5min at room temperature. Fixed testes were washed 2x in PBS/PIC by vortexing and centrifuging at 500rpm for 5min at 4°C, and 1x in 1ml PBS/PIC on a rotator for 10min at 4°C. Testes were centrifuged at 500rpm for 5min at 4°C, flash-frozen and stored at -80°C. On day of ChIP, fixed testes were thawed on ice and resuspended in 1ml 1x Lysis Buffer/PIC, homogenized with a plastic pestle that fits a 1.5ml eppendorf tube, and passed through a 35nm cell strainer. Cells were lysed on ice for 30min, and the nuclei were pelleted by centrifugation at 500rpm for 5min at 4°C. The supernatant was removed, and nuclei were resuspended in 300μl RIPA/PIC and incubated on ice 20 min. Chromatin was sonicated to 100-500bp fragments in a Biorupter for 3 rounds of 15min on High, 30s on/off. Debris was pelleted at 14,000rpm for 20min at 4°C. 10% of sheared chromatin was taken as input. 15–20μg of sheared chromatin were incubated overnight with 25μl protG Dynabeads (Thermo Fisher 10004D) pre-incubated for 3hrs with 5μl anti-CBX2 (Bethyl #A302-524A) or anti-IgG (CST #2729S). 50 E13.5 and E14.5 testes were pooled for each ChIP. Beads were washed 2x in 200μl of ice-cold RIPA, 1x in 200μl ice-cold RIPA/High Salt, 1x in 200μl ice-cold LiCl buffer and 1x in 100μl of TE by moving tubes from the front of the magnet to the back twice. Washed beads were resuspended in 100ul Elution Buffer. Beads and input were then incubated with 0.5μl of RNase Cocktail and 0.5ul of protK (10mg/ml) at 37°C for 30min and 63°C for 4 hours to reverse crosslinking. DNA was purified using a MinElute Qiagen Kit (#28004) and used for qPCR. qPCR was analyzed as % Input.

#### ChIP-re-ChIP

4 adult testes per experiment were processed, fixed, and chromatin was sheared as described above (buffers from [37]). Prior to IP, sheared chromatin was divided into three tubes and each was incubated overnight at 4°C on an end-over-end rotator with 25μl of protG Dynabeads (Thermo Fisher 10004D) pre-incubated with 5μl anti-H3K27me3 (CST #9733S). 10% of the input was kept and stored at -20°C for later. The next day, beads were washed 6x in ChIP Lysis Buffer/PIC and 1x in ice-cold TE. Chromatin was eluted from the beads by first incubating in 25μl of 100mM DTT for 5 minutes at room temperature, and then with an additional 25μl of Chromatin Release Buffer (500mM NaCl, 2% SDS, 2% DOC, 2x PIC) for 30 minutes at 37°C. The eluted chromatin was pooled and diluted 4x with Dilution Buffer. The sample was then concentrated with a 50k Amicon Ultra 0.5ml centrifugal filter (Sigma-Aldrich #UFC505024) to remove SDS and DTT from the buffer. Sample was then brought up to 900μl in Dilution Buffer/PIC and divided into two tubes. One was incubated with 25μl of protG Dynabeads (Thermo Fisher 10004D) pre-incubated with 5μl of anti-H3K4me3 (Active Motif #39159), and the other was incubated in beads with no antibody as a control overnight at 4°C on an end-over-end rotator. The next day, beads were washed 2x in 200μl of ice-cold RIPA, 1x in 200μl ice-cold RIPA/High Salt, 1x in 200μl ice-cold LiCl buffer and 1x in 100μl of TE by moving tubes from the front of the magnet to the back twice. Washed beads were resuspended in 100μl Elution Buffer (20mM Tris-HCl pH7.5, 5mM EDTA, 50mM NaCl, 1%SDS) with 0.5μl of RNase Cocktail (Invitrogen AM2286) and 0.5μl of protK (10mg/ml) and incubated at 37°C for 30min then 63°C for 1 hour. DNA was purified using a MinElute Qiagen Kit (28004) and used for qPCR. qPCR was analyzed as % Input.

#### Ethics statement

All animal studies were performed under IACUC compliance (protocol # 00003863). Euthanasia was performed by CO2 administration followed by cervical dislocation.

## Supporting information

S1 Fig Histone marks are consistent between biological replicates and validated by ChIP-qPCR. (A) ChIP-seq tracks for H3 (black), H3K27me3 (red) and H3K4me3 (green) for both biological replicates are shown side by side in XY and XX, E10.5 and E13.5 purified supporting cells. (B&C) ChIP-qPCR validation of ChIP-seq for H3K27me3 (red) and H3K4me3 (green) in FACS-purified E10.5 XY and XX cells (B) and E13.5 XY and XX (C). Each ChIP-qPCR was performed on 3 biological replicates, each replicate contained pooled cells from several gonads. ChIP-seq tracks for depicted genes are in Figs 2 & 3. Values represent mean ± SEM.
(TIF)

S2 Fig Epigenetic profiling of supporting cells during sex determination. (A) Percentage of total number of promoters annotated in the mm9 genome marked by H3K27me3-only (red), both H3K27me3 and H3K4me3 (peach), H3K4me3-only (green), or neither (grey). (B) Boxplots of gene expression values (log intensity value from Nef et al., 2005) for H3K4me3-only promoters, H3K4me3 and H3K27me3 promoters (bivalent) or H3K27me3-only promoters, in XY (blue) or XX (pink) supporting cells at E10.5 (left) and E13.5 (right) (outliers excluded). *** represents p<0.0001 as determined by student’s t test. (C&D) Venn diagrams depicting number of overlapping bivalent promoters between Sertoli cells (blue) and pregranulosa cells (pink) at E10.5 (C) and E13.5 (D).
(TIF)

S3 Fig Up-regulation of testis or ovary pathway genes is associated with loss of H3K27me3. (A) Bar graphs denoting gene expression log intensity values from Nef et al, 2005, for select genes in XY (blue) and XX (pink) supporting cells at E13.5. *** represents p<0.0001 as determined by student’s t test. Values represent mean ± SEM. (B) A closer look at H3K27me3 ChIP-seq tracks at *Foxl2* in E13.5 pregranulosa cells (top) and Sertoli cells (bottom) shows loss of H3K27me3 at the promoter of *Foxl2* in XX but not XY cells.
(TIF)

S4 Fig Repressed ovary pathway genes retain bivalent marks in adult Sertoli cells. (A) ChIP-qPCR for H3K27me3 (red), H3K4me3 (green) and IgG (grey) at the promoter of several ovary-specific genes in purified Sertoli cells from adult (>2m/o) males. Each qPCR was performed on 3 biological replicates, each replicate contained purified Sertoli cells from 1–2 adult males. (B) ChIP-re-ChIP on adult testes for H3K27me3 followed by either H3K4me3, or a no-antibody control, performed on two independent replicates, with testes from 1–2 adult males. Values represent mean ± SEM.
(TIF)

S5 Fig H3K27me3 spreads over repressed loci in Sertoli cells. UCSC genome browser tracks of example repressed genes in XY supporting cells where H3K27me3 deposition (red) is confined to narrow regions at E10.5 (top rows) and spreads upstream and downstream of the TSS, and over the gene body at E13.5 (bottom rows). Collapsed H3K27me3 tracks are represented in bars above tracks.
(TIF)

S6 Fig Pregranulosa-determining genes with critical roles in ovary development are targets of PcG repression in Sertoli cells. Heatmap of H3K27me3 enrichment levels at the promoters of pregranulosa-promoting genes in Sertoli cells, ranging from high (light red) to low (light green). Values represent log2 enrichment normalized to H3. A closer look at the genes with >4 H3K27me3 enrichment are shown in the right column. Genes with known roles in ovary development are bolded.
(TIF)

S7 Fig The Wnt pathway is targeted for H3K27me3-mediated repression in Sertoli cells. (A&B) Gene Ontology functional analysis using GREAT of pregranulosa-specific promoters and flanking regions marked by H3K27me3 shows that the Wnt signaling pathway is significantly targeted for repression in Sertoli cells (A), and that the developmental processes most highly represented are those associated with the formation of the reproductive system, in particular the female reproductive and urogenital system (B). (C) Genome browser tracks showing ChIP-seq profiles for H3K4me3 (green) and H3K27me3 (red). Promoters highlighted in blue. Black boxes represent significant enrichment when compared to flanking regions as determined by HOMER.
(TIF)

S8 Fig Sex reversal is rescued in *Cbx2;Wnt4* double knockout XY gonads. (A&B) XY gonads are stained with the pregranulosa cell marker FOXL2 (green), Sertoli cell marker SOX9 (red), and vasculature and germ cell marker PECAM (blue). Loss of *Cbx2* in E13.5 XY gonads leads to reduction of SOX9+ Sertoli cells, gain of FOXL2+ pregranulosa cells, and testis cords are lost (A). *Cbx2*^*-/-*^*;Wnt4*^*-/-*^ DKO gonads do not have FOXL2+ pregranulosa cells, and testis cord formation is rescued (B). XY *Cbx2*^*-/-*^ gonads develop as ovaries (C). DKO gonads develop as testes (D).
(TIF)

S9 Fig CBX2 targets *Lef1* for repression in adult testes. ChIP-qPCR for CBX2 adult testes from >2m/o mice (2 males/experiment). * represents p<0.01 as determined by student’s t test when compared to the negative control *Gapdh*. Values represent mean ± SEM.
(TIF)

S1 Table ChIP-qPCR primers. (DOCX)

S2 Table Genotyping primers. (DOCX)

S3 Table Immunofluorescence antibodies. (DOCX)

S4 Table ChIP antibodies. (DOCX)

S1 File Complete list of Sertoli-specific and pregranulosa-specific H3K27me3+, H3K4me3+ and bivalent genes and their genomic coordinates (mm9) in XX and XY supporting cells at E10.5 and E13.5. (XLSX)

## References

[1] Albrecht K.H. and Eicher E.M., Evidence that Sry is expressed in pre-Sertoli cells and Sertoli and granulosa cells have a common precursor. Dev Biol, 2001 240(1): p. 92–107. 10.1006/dbio.2001.0438 11784049

[2] Burgoyne P.S., et al, The genetic basis of XX-XY differences present before gonadal sex differentiation in the mouse. Philos Trans R Soc Lond B Biol Sci, 1995 350(1333): p. 253–60 discussion 260–1. 10.1098/rstb.1995.0159 8570689

[3] Sinclair A.H., et al, A gene from the human sex-determining region encodes a protein with homology to a conserved DNA-binding motif. Nature, 1990 346(6281): p. 240–4. 10.1038/346240a0 1695712

[4] Gubbay J., et al, A gene mapping to the sex-determining region of the mouse Y chromosome is a member of a novel family of embryonically expressed genes. Nature, 1990 346(6281): p. 245–50. 10.1038/346245a0 2374589

[5] Koopman P., et al, Male development of chromosomally female mice transgenic for Sry. Nature, 1991 351(6322): p. 117–21. 10.1038/351117a0 2030730

[6] Bullejos M. and Koopman P., Spatially dynamic expression of Sry in mouse genital ridges. Dev Dyn, 2001 221(2): p. 201–5. 10.1002/dvdy.1134 11376487

[7] Hacker A., et al, Expression of Sry, the mouse sex determining gene. Development, 1995 121(6): p. 1603–14. 7600978 10.1242/dev.121.6.1603

[8] Sekido R., et al, SOX9 is up-regulated by the transient expression of SRY specifically in Sertoli cell precursors. Dev Biol, 2004 274(2): p. 271–9. 10.1016/j.ydbio.2004.07.011 15385158

[9] Kim Y., et al, Fgf9 and Wnt4 act as antagonistic signals to regulate mammalian sex determination. PLoS Biol, 2006 4(6): p. e187 10.1371/journal.pbio.0040187 16700629 PMC1463023

[10] Maatouk D.M., et al, Stabilization of beta-catenin in XY gonads causes male-to-female sex-reversal. Hum Mol Genet, 2008 17(19): p. 2949–55. 10.1093/hmg/ddn193 18617533 PMC2536503

[11] Ottolenghi C., et al, Loss of Wnt4 and Foxl2 leads to female-to-male sex reversal extending to germ cells. Hum Mol Genet, 2007 16(23): p. 2795–804. 10.1093/hmg/ddm235 17728319

[12] Jameson S.A., Lin Y.T., and Capel B., Testis development requires the repression of Wnt4 by Fgf signaling. Dev Biol, 2012 370(1): p. 24–32. 10.1016/j.ydbio.2012.06.009 22705479 PMC3634333

[13] Munger S.C., et al, Fine time course expression analysis identifies cascades of activation and repression and maps a putative regulator of mammalian sex determination. PLoS Genet, 2013 9(7): p. e1003630 10.1371/journal.pgen.1003630 23874228 PMC3708841

[14] Jameson S.A., et al, Temporal transcriptional profiling of somatic and germ cells reveals biased lineage priming of sexual fate in the fetal mouse gonad. PLoS Genet, 2012 8(3): p. e1002575 10.1371/journal.pgen.1002575 22438826 PMC3305395

[15] Uhlenhaut N.H., et al, Somatic sex reprogramming of adult ovaries to testes by FOXL2 ablation. Cell, 2009 139(6): p. 1130–42. 10.1016/j.cell.2009.11.021 20005806

[16] Matson C.K., et al, DMRT1 prevents female reprogramming in the postnatal mammalian testis. Nature, 2011 476(7358): p. 101–4. 10.1038/nature10239 21775990 PMC3150961

[17] Kuroki S., et al, Epigenetic regulation of mouse sex determination by the histone demethylase Jmjd1a. Science, 2013 341(6150): p. 1106–9. 10.1126/science.1239864 24009392

[18] Kuroki S., et al, Rescuing the aberrant sex development of H3K9 demethylase Jmjd1a-deficient mice by modulating H3K9 methylation balance. PLoS Genet, 2017 13(9): p. e1007034 10.1371/journal.pgen.1007034 28949961 PMC5630185

[19] Carre G.A., et al, Loss of p300 and CBP disrupts histone acetylation at the mouse Sry promoter and causes XY gonadal sex reversal. Hum Mol Genet, 2017. 10.1093/hmg/ddx398 PMC5886154 29145650

[20] Simon J.A. and Kingston R.E., Mechanisms of polycomb gene silencing: knowns and unknowns. Nat Rev Mol Cell Biol, 2009 10(10): p. 697–708. 10.1038/nrm2763 19738629

[21] Cao R., et al, Role of histone H3 lysine 27 methylation in Polycomb-group silencing. Science, 2002 298(5595): p. 1039–43. 10.1126/science.1076997 12351676

[22] Shen X., et al, EZH1 mediates methylation on histone H3 lysine 27 and complements EZH2 in maintaining stem cell identity and executing pluripotency. Mol Cell, 2008 32(4): p. 491–502. 10.1016/j.molcel.2008.10.016 19026780 PMC2630502

[23] Lau M.S., et al, Mutation of a nucleosome compaction region disrupts Polycomb-mediated axial patterning. Science, 2017 355(6329): p. 1081–1084. 10.1126/science.aah5403 28280206 PMC5503153

[24] Blackledge N.P., et al, Variant PRC1 complex-dependent H2A ubiquitylation drives PRC2 recruitment and polycomb domain formation. Cell, 2014 157(6): p. 1445–59. 10.1016/j.cell.2014.05.004 24856970 PMC4048464

[25] Cooper S., et al, Targeting polycomb to pericentric heterochromatin in embryonic stem cells reveals a role for H2AK119u1 in PRC2 recruitment. Cell Rep, 2014 7(5): p. 1456–1470. 10.1016/j.celrep.2014.04.012 24857660 PMC4062935

[26] Santos-Rosa H., et al, Active genes are tri-methylated at K4 of histone H3. Nature, 2002 419(6905): p. 407–11. 10.1038/nature01080 12353038

[27] Bernstein B.E., et al, A bivalent chromatin structure marks key developmental genes in embryonic stem cells. Cell, 2006 125(2): p. 315–26. 10.1016/j.cell.2006.02.041 16630819

[28] Azuara V., et al, Chromatin signatures of pluripotent cell lines. Nat Cell Biol, 2006 8(5): p. 532–8. 10.1038/ncb1403 16570078

[29] Pan G., et al, Whole-genome analysis of histone H3 lysine 4 and lysine 27 methylation in human embryonic stem cells. Cell Stem Cell, 2007 1(3): p. 299–312. 10.1016/j.stem.2007.08.003 18371364

[30] Voigt P., Tee W.W., and Reinberg D., A double take on bivalent promoters. Genes Dev, 2013 27(12): p. 1318–38. 10.1101/gad.219626.113 23788621 PMC3701188

[31] Katoh-Fukui Y., et al, Male-to-female sex reversal in M33 mutant mice. Nature, 1998 393(6686): p. 688–92. 10.1038/31482 9641679

[32] Biason-Lauber A., et al, Ovaries and female phenotype in a girl with 46,XY karyotype and mutations in the CBX2 gene. Am J Hum Genet, 2009 84(5): p. 658–63. 10.1016/j.ajhg.2009.03.016 19361780 PMC2680992

[33] Katoh-Fukui Y., et al, Cbx2, a polycomb group gene, is required for Sry gene expression in mice. Endocrinology, 2012 153(2): p. 913–24. 10.1210/en.2011-1055 22186409

[34] Harris A., et al, ZNRF3 functions in mammalian sex determination by inhibiting canonical WNT signaling. Proc Natl Acad Sci U S A, 2018 115(21): p. 5474–5479. 10.1073/pnas.1801223115 29735715 PMC6003506

[35] Colvin J.S., et al, Male-to-female sex reversal in mice lacking fibroblast growth factor 9. Cell, 2001 104(6): p. 875–89. 11290325 10.1016/s0092-8674(01)00284-7

[36] Bagheri-Fam S., et al, Loss of Fgfr2 leads to partial XY sex reversal. Dev Biol, 2008 314(1): p. 71–83. 10.1016/j.ydbio.2007.11.010 18155190

[37] van Galen P., et al, A Multiplexed System for Quantitative Comparisons of Chromatin Landscapes. Mol Cell, 2016 61(1): p. 170–80. 10.1016/j.molcel.2015.11.003 26687680 PMC4707994

[38] Beverdam A. and Koopman P., Expression profiling of purified mouse gonadal somatic cells during the critical time window of sex determination reveals novel candidate genes for human sexual dysgenesis syndromes. Hum Mol Genet, 2006 15(3): p. 417–31. 10.1093/hmg/ddi463 16399799

[39] Sekido R. and Lovell-Badge R., Sex determination involves synergistic action of SRY and SF1 on a specific Sox9 enhancer. Nature, 2008 453(7197): p. 930–4. 10.1038/nature06944 18454134

[40] Gonen N., et al, Sex reversal following deletion of a single distal enhancer of Sox9. Science, 2018. 10.1126/science.aas9408 PMC6034650 29903884

[41] Nef S., et al, Gene expression during sex determination reveals a robust female genetic program at the onset of ovarian development. Dev Biol, 2005 287(2): p. 361–77. 10.1016/j.ydbio.2005.09.008 16214126

[42] Xu C.R., et al, Chromatin "prepattern" and histone modifiers in a fate choice for liver and pancreas. Science, 2011 332(6032): p. 963–6. 10.1126/science.1202845 21596989 PMC3128430

[43] Pinter S.F., et al, Spreading of X chromosome inactivation via a hierarchy of defined Polycomb stations. Genome Res, 2012 22(10): p. 1864–76. 10.1101/gr.133751.111 22948768 PMC3460182

[44] Behrens J., et al, Functional interaction of beta-catenin with the transcription factor LEF-1. Nature, 1996 382(6592): p. 638–42. 10.1038/382638a0 8757136

[45] Jacob E., et al, Dual function of polycomb group proteins in differentiated murine T helper (CD4+) cells. J Mol Signal, 2011 6: p. 5 10.1186/1750-2187-6-5 21624129 PMC3127800

[46] Herz H.M., et al, Polycomb repressive complex 2-dependent and -independent functions of Jarid2 in transcriptional regulation in Drosophila. Mol Cell Biol, 2012 32(9): p. 1683–93. 10.1128/MCB.06503-11 22354997 PMC3347239

[47] Mousavi K., et al, Polycomb protein Ezh1 promotes RNA polymerase II elongation. Mol Cell, 2012 45(2): p. 255–62. 10.1016/j.molcel.2011.11.019 22196887 PMC12310276

[48] Garcia-Moreno S.A., et al, Gonadal supporting cells acquire sex-specific chromatin landscapes during mammalian sex determination. Dev Biol, 2019 446(2): p. 168–179. 10.1016/j.ydbio.2018.12.023 30594505 PMC6368449

[49] Hiramatsu R., et al, A critical time window of Sry action in gonadal sex determination in mice. Development, 2009 136(1): p. 129–38. 10.1242/dev.029587 19036799

[50] Kashimada K. and Koopman P., Sry: the master switch in mammalian sex determination. Development, 2010 137(23): p. 3921–30. 10.1242/dev.048983 21062860

[51] Akiyama H., et al, Interactions between Sox9 and beta-catenin control chondrocyte differentiation. Genes Dev, 2004 18(9): p. 1072–87. 10.1101/gad.1171104 15132997 PMC406296

[52] Sellak H., Wu S., and Lincoln T.M., KLF4 and SOX9 transcription factors antagonize beta-catenin and inhibit TCF-activity in cancer cells. Biochim Biophys Acta, 2012 1823(10): p. 1666–75. 10.1016/j.bbamcr.2012.06.027 22766303 PMC3633466

[53] Rahmoun M., et al, In mammalian foetal testes, SOX9 regulates expression of its target genes by binding to genomic regions with conserved signatures. Nucleic Acids Res, 2017 45(12): p. 7191–7211. 10.1093/nar/gkx328 28472341 PMC5499551

[54] Nicol B., et al, Genome-wide identification of FOXL2 binding and characterization of FOXL2 feminizing action in the fetal gonads. Hum Mol Genet, 2018 27(24): p. 4273–4287. 10.1093/hmg/ddy312 30212841 PMC6276834

[55] Vainio S., et al, Female development in mammals is regulated by Wnt-4 signalling. Nature, 1999 397(6718): p. 405–9. 10.1038/17068 9989404

[56] Maatouk D.M., et al, Genome-wide identification of regulatory elements in Sertoli cells. Development, 2017 144(4): p. 720–730. 10.1242/dev.142554 28087634 PMC5312035

[57] Anway M.D., et al, Isolation of sertoli cells from adult rat testes: an approach to ex vivo studies of Sertoli cell function. Biol Reprod, 2003 68(3): p. 996–1002. 10.1095/biolreprod.102.008045 12604653

