# Supplementary figures and images for: CBX2 is required to stabilize the testis pathway by repressing Wnt signaling
Source: PLoS Genet. 2019 May 22;15(5):e1007895. doi: 10.1371/journal.pgen.1007895 (PMC6548405; doi:10.1371/journal.pgen.1007895)

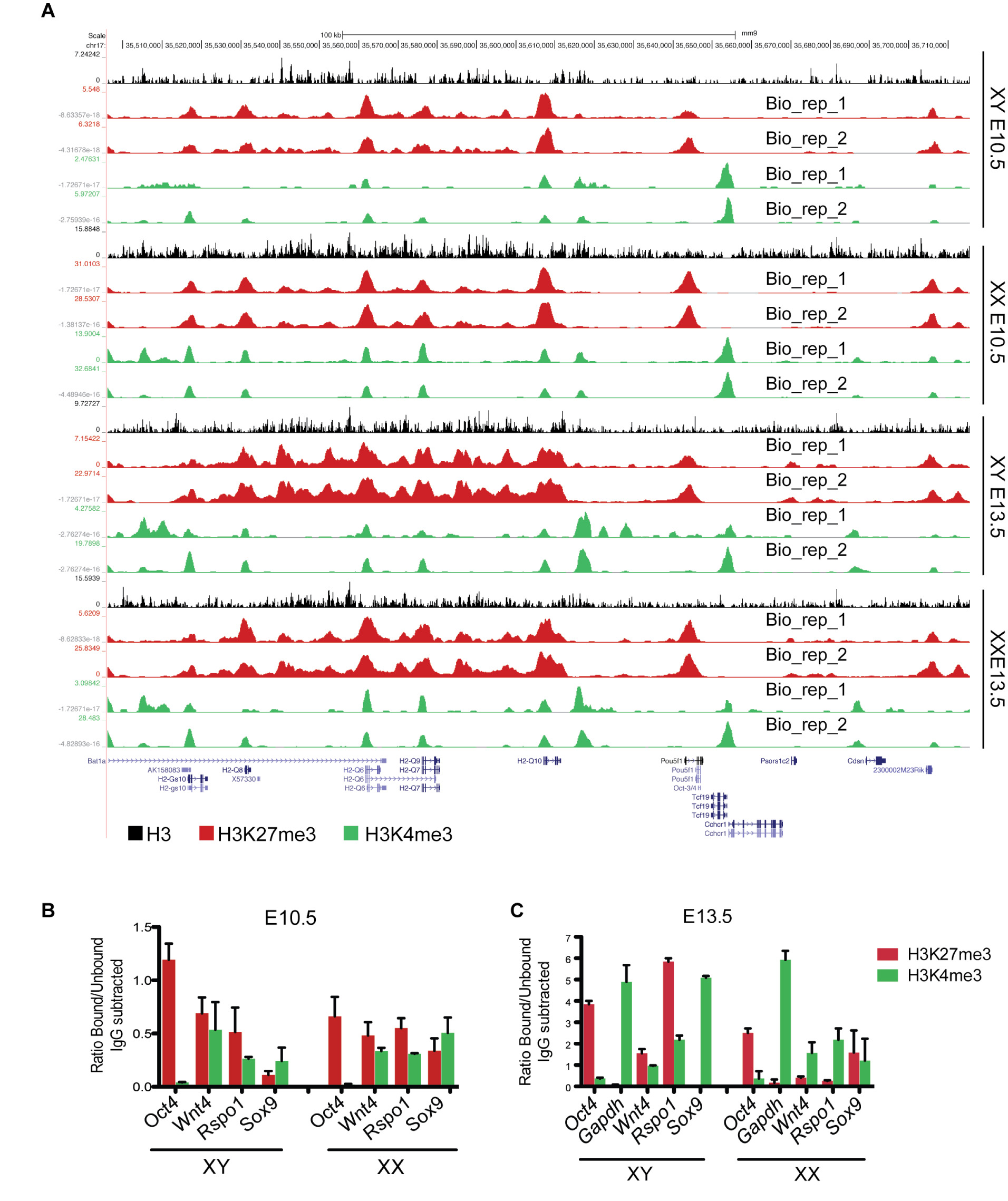

Supplement: S1 Fig — (A) ChIP-seq tracks for H3 (black), H3K27me3 (red) and H3K4me3 (green) for both biological replicates are shown side by side in XY and XX, E10.5 and E13.5 purified supporting cells. (B&C) ChIP-qPCR validation of ChIP-seq for H3K27me3 (red) and H3K4me3 (green) in FACS-purified E10.5 XY and XX cells (B) and E13.5 XY and XX (C). Each ChIP-qPCR was performed on 3 biological replicates, each replicate contained pooled cells from several gonads. ChIP-seq tracks for depicted genes are in Figs 2 & 3. Values represent mean ± SEM. (TIF) [file pgen.1007895.s001.tif]

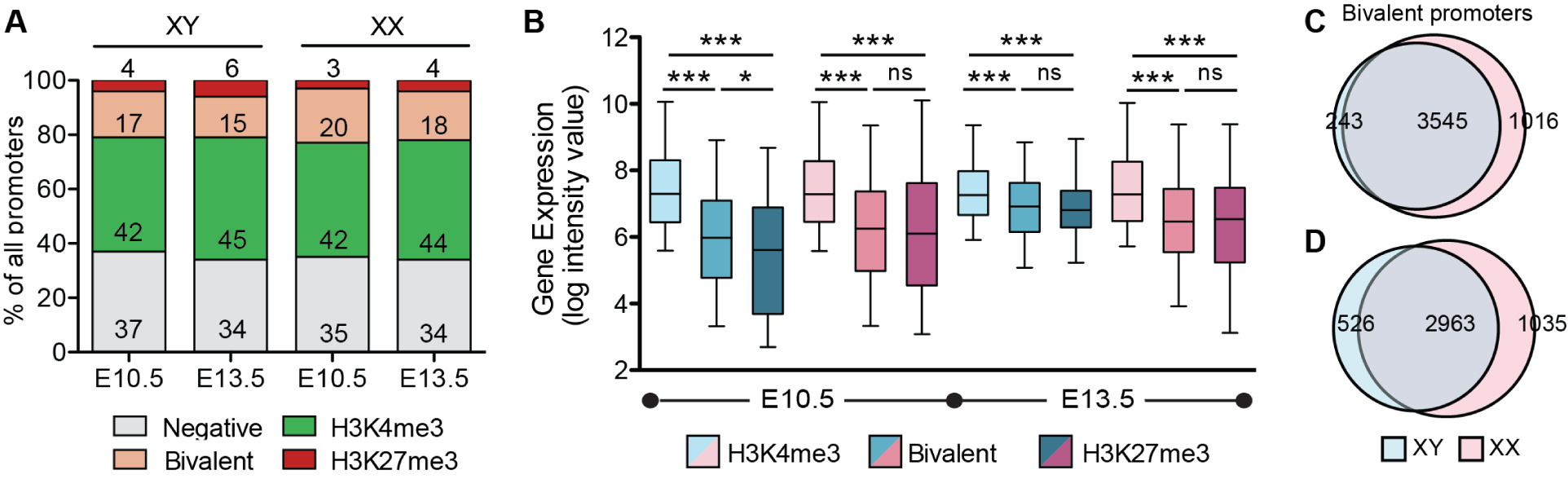

Supplement: S2 Fig — (A) Percentage of total number of promoters annotated in the mm9 genome marked by H3K27me3-only (red), both H3K27me3 and H3K4me3 (peach), H3K4me3-only (green), or neither (grey). (B) Boxplots of gene expression values (log intensity value from Nef et al., 2005) for H3K4me3-only promoters, H3K4me3 and H3K27me3 promoters (bivalent) or H3K27me3-only promoters, in XY (blue) or XX (pink) supporting cells at E10.5 (left) and E13.5 (right) (outliers excluded). *** represents p<0.0001 as determined by student’s t test. (C&D) Venn diagrams depicting number of overlapping bivalent promoters between Sertoli cells (blue) and pregranulosa cells (pink) at E10.5 (C) and E13.5 (D). (TIF) [file pgen.1007895.s002.tif]

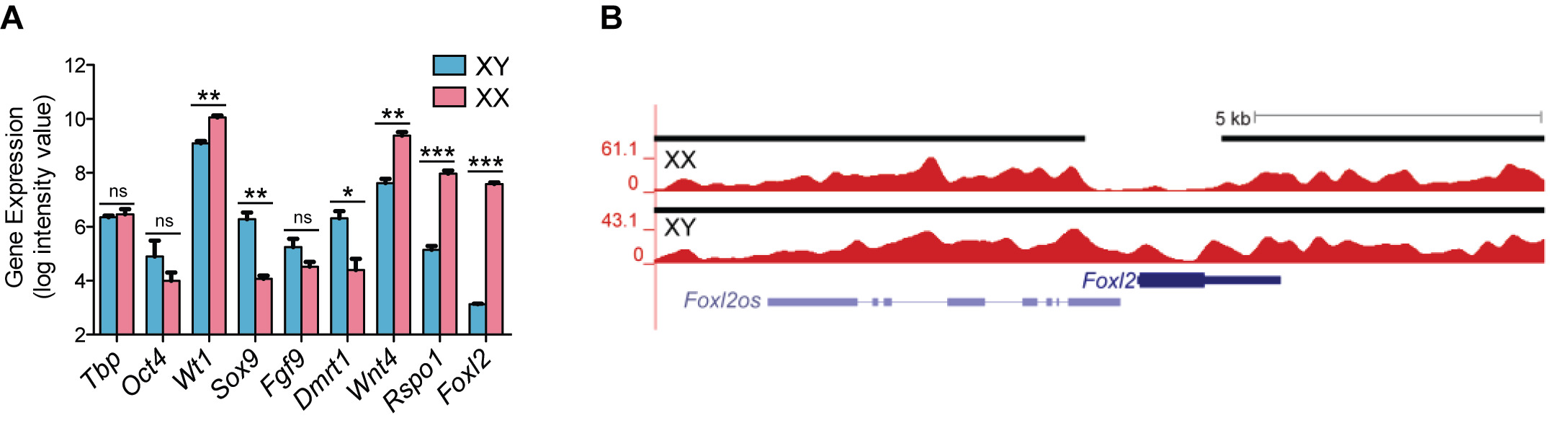

Supplement: S3 Fig — (A) Bar graphs denoting gene expression log intensity values from Nef et al, 2005, for select genes in XY (blue) and XX (pink) supporting cells at E13.5. *** represents p<0.0001 as determined by student’s t test. Values represent mean ± SEM. (B) A closer look at H3K27me3 ChIP-seq tracks at Foxl2 in E13.5 pregranulosa cells (top) and Sertoli cells (bottom) shows loss of H3K27me3 at the promoter of Foxl2 in XX but not XY cells. (TIF) [file pgen.1007895.s003.tif]

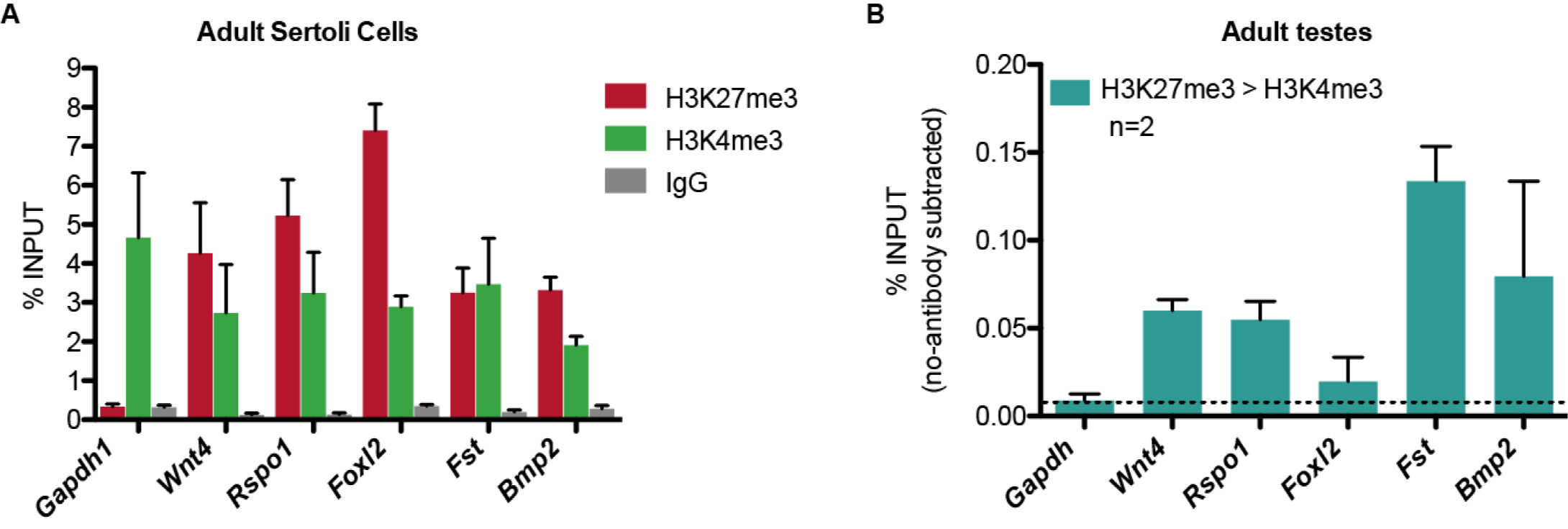

Supplement: S4 Fig — (A) ChIP-qPCR for H3K27me3 (red), H3K4me3 (green) and IgG (grey) at the promoter of several ovary-specific genes in purified Sertoli cells from adult (>2m/o) males. Each qPCR was performed on 3 biological replicates, each replicate contained purified Sertoli cells from 1–2 adult males. (B) ChIP-re-ChIP on adult testes for H3K27me3 followed by either H3K4me3, or a no-antibody control, performed on two independent replicates, with testes from 1–2 adult males. Values represent mean ± SEM. (TIF) [file pgen.1007895.s004.tif]

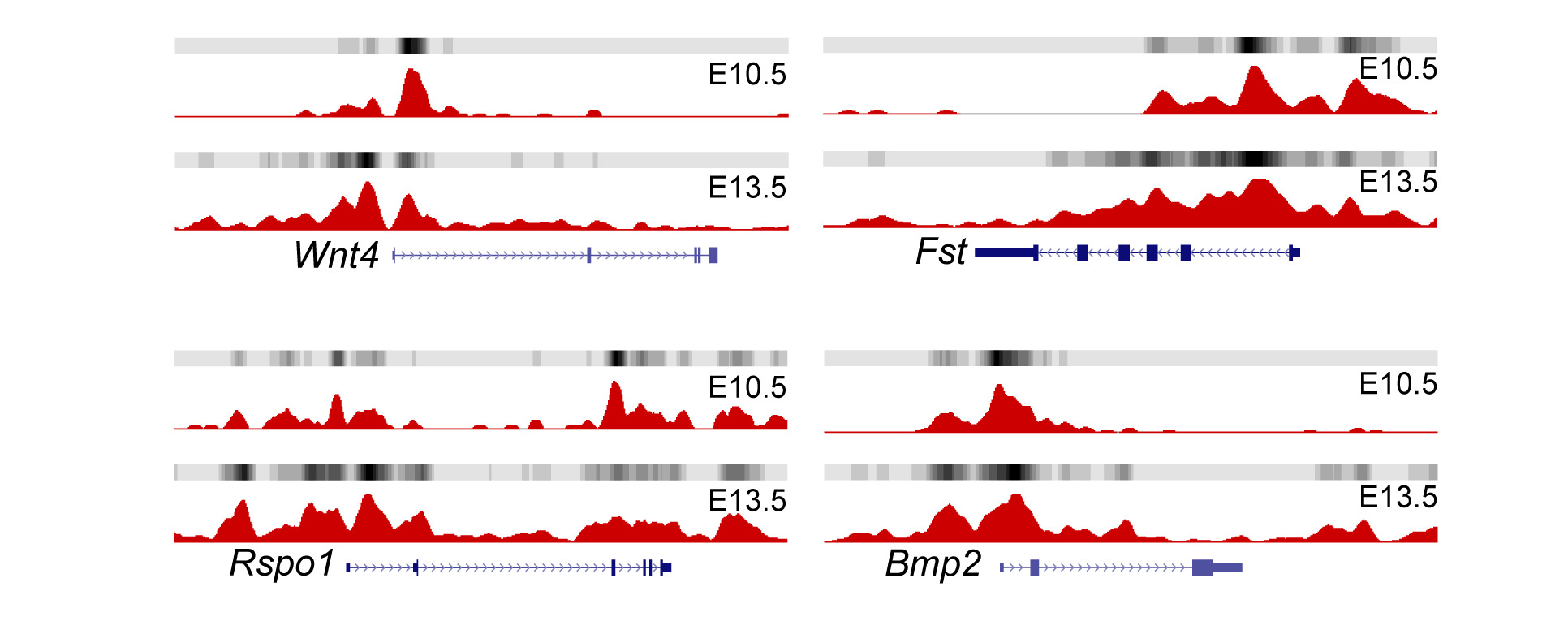

Supplement: S5 Fig — UCSC genome browser tracks of example repressed genes in XY supporting cells where H3K27me3 deposition (red) is confined to narrow regions at E10.5 (top rows) and spreads upstream and downstream of the TSS, and over the gene body at E13.5 (bottom rows). Collapsed H3K27me3 tracks are represented in bars above tracks. (TIF) [file pgen.1007895.s005.tif]

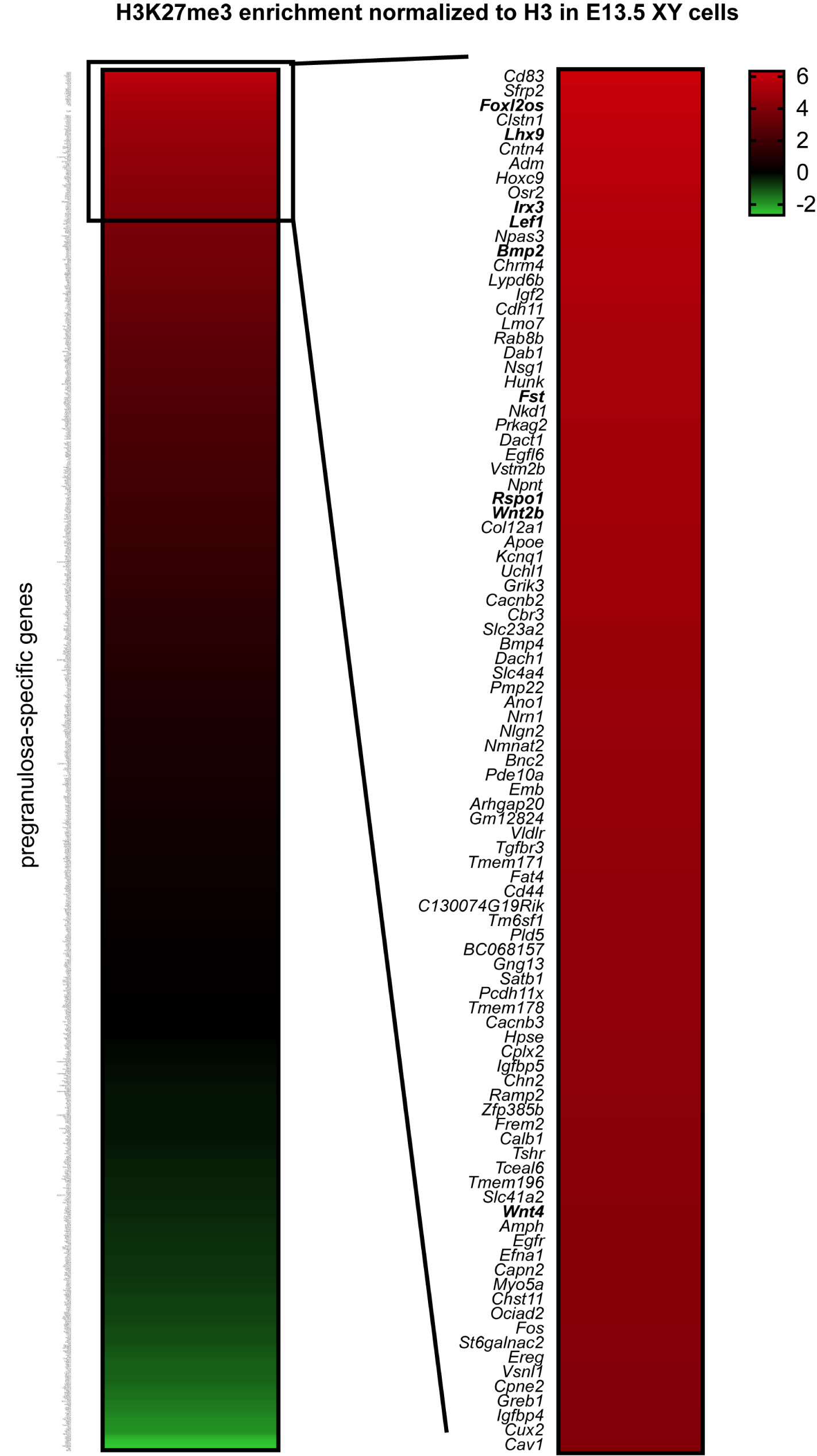

Supplement: S6 Fig — Heatmap of H3K27me3 enrichment levels at the promoters of pregranulosa-promoting genes in Sertoli cells, ranging from high (light red) to low (light green). Values represent log2 enrichment normalized to H3. A closer look at the genes with >4 H3K27me3 enrichment are shown in the right column. Genes with known roles in ovary development are bolded. (TIF) [file pgen.1007895.s006.tif]

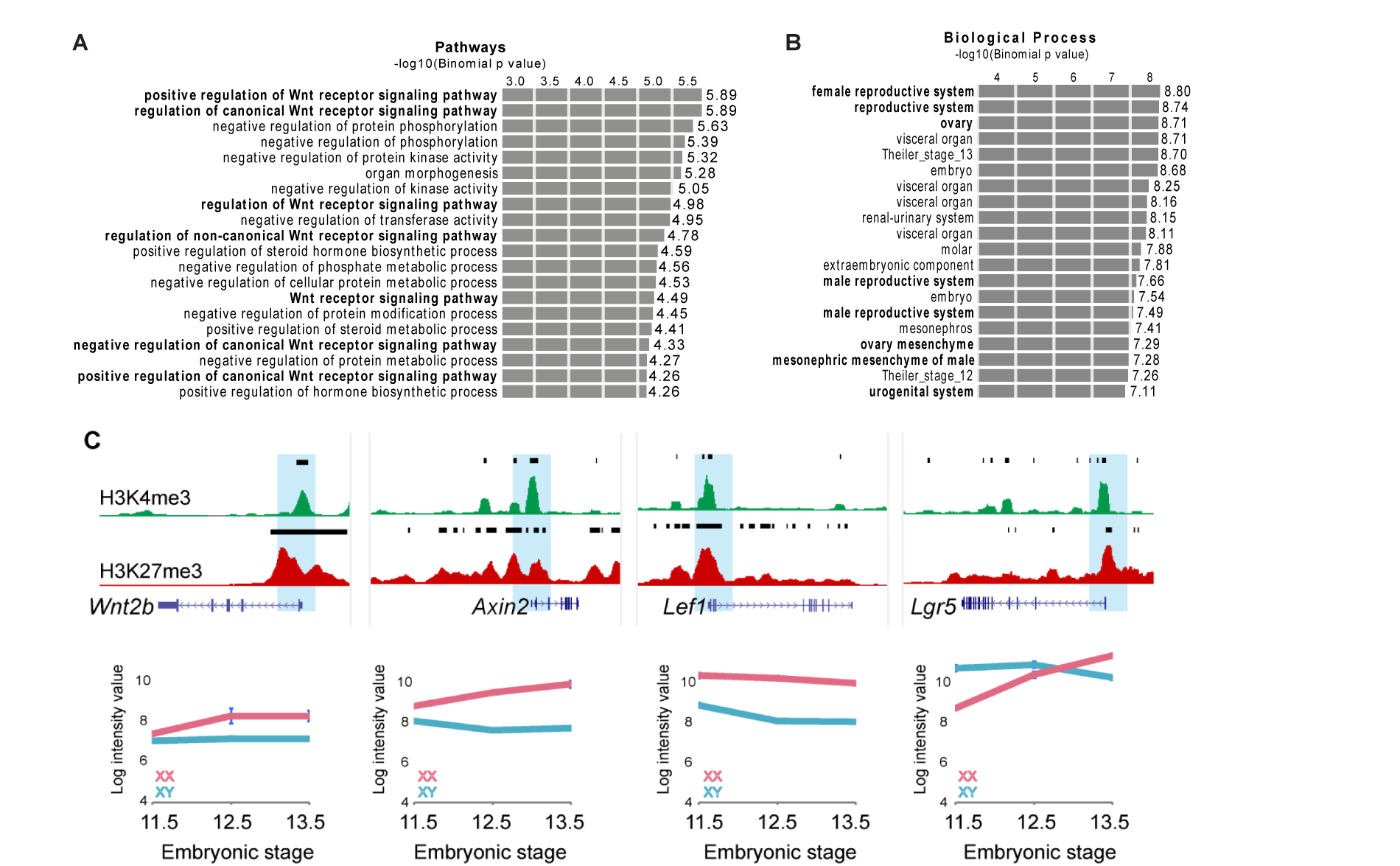

Supplement: S7 Fig — (A&B) Gene Ontology functional analysis using GREAT of pregranulosa-specific promoters and flanking regions marked by H3K27me3 shows that the Wnt signaling pathway is significantly targeted for repression in Sertoli cells (A), and that the developmental processes most highly represented are those associated with the formation of the reproductive system, in particular the female reproductive and urogenital system (B). (C) Genome browser tracks showing ChIP-seq profiles for H3K4me3 (green) and H3K27me3 (red). Promoters highlighted in blue. Black boxes represent significant enrichment when compared to flanking regions as determined by HOMER. (TIF) [file pgen.1007895.s007.tif]

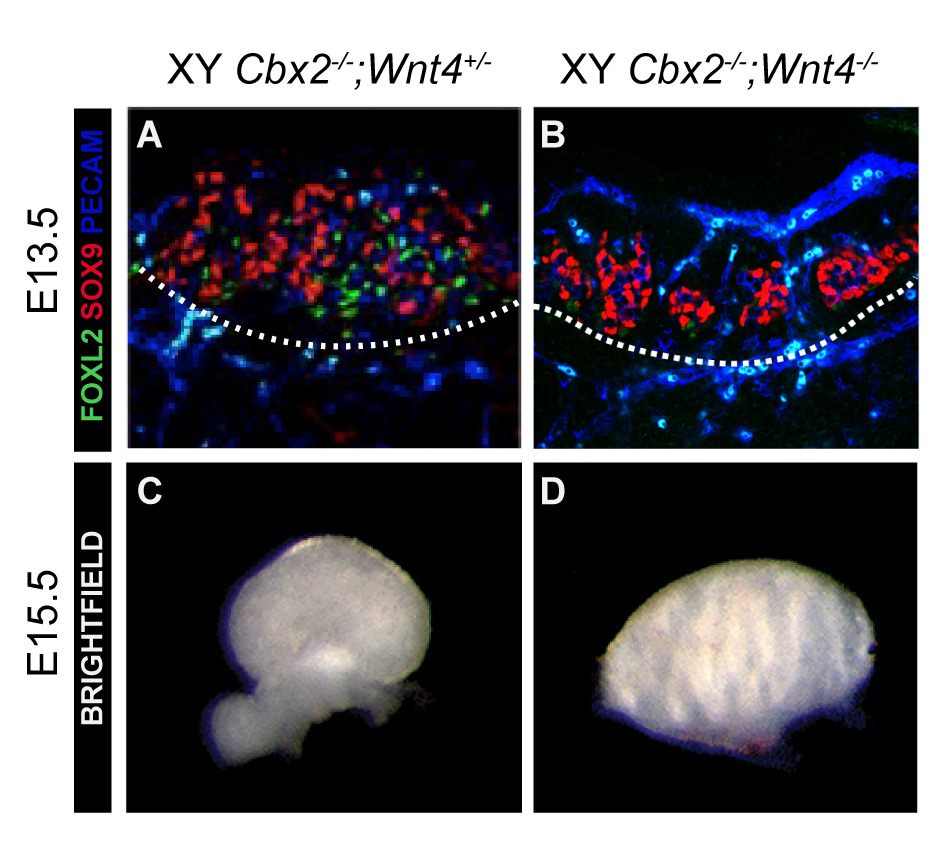

Supplement: S8 Fig — (A&B) XY gonads are stained with the pregranulosa cell marker FOXL2 (green), Sertoli cell marker SOX9 (red), and vasculature and germ cell marker PECAM (blue). Loss of Cbx2 in E13.5 XY gonads leads to reduction of SOX9+ Sertoli cells, gain of FOXL2+ pregranulosa cells, and testis cords are lost (A). Cbx2-/-;Wnt4-/- DKO gonads do not have FOXL2+ pregranulosa cells, and testis cord formation is rescued (B). XY Cbx2-/- gonads develop as ovaries (C). DKO gonads develop as testes (D). (TIF) [file pgen.1007895.s008.tif]

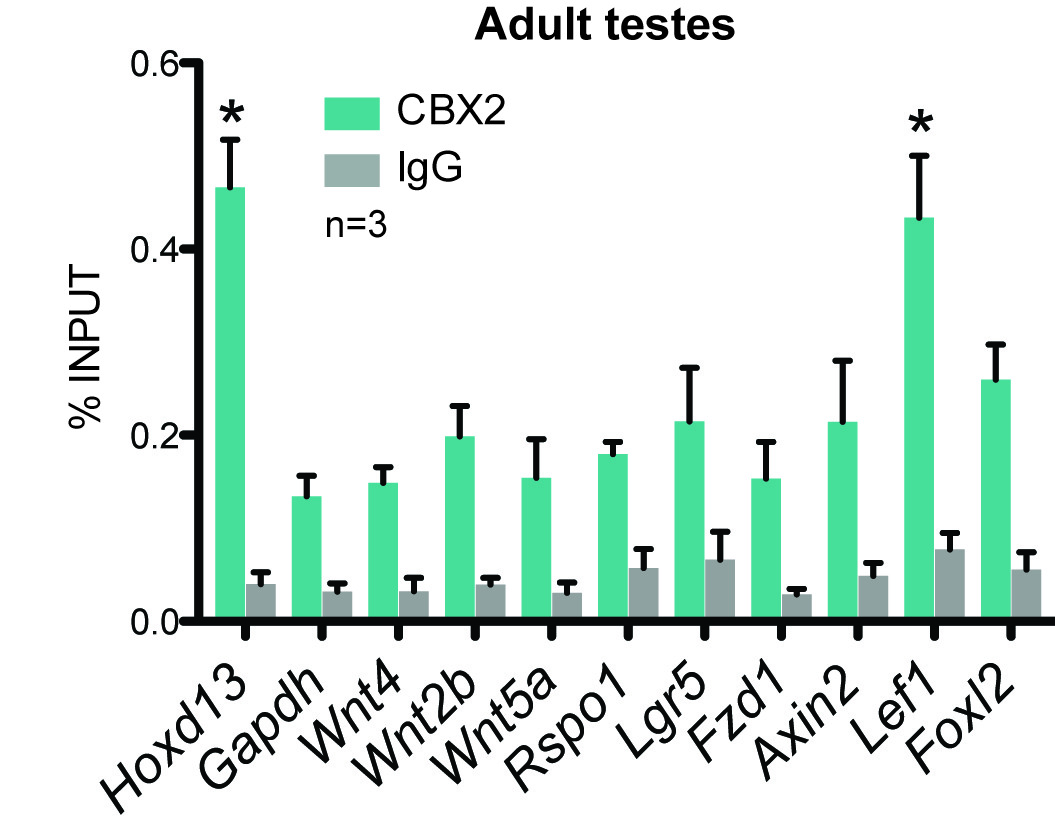

Supplement: S9 Fig — ChIP-qPCR for CBX2 adult testes from >2m/o mice (2 males/experiment). * represents p<0.01 as determined by student’s t test when compared to the negative control Gapdh. Values represent mean ± SEM. (TIF) [file pgen.1007895.s009.tif]
