# Supplementary material for: CBX2 is required to stabilize the testis pathway by repressing Wnt signaling
Source: PLoS Genet. 2019 May 22;15(5):e1007895. doi: 10.1371/journal.pgen.1007895 (PMC6548405; doi:10.1371/journal.pgen.1007895)
Supplement: S1 Table — (DOCX) [file pgen.1007895.s010.docx]

| **Gene** | **Forward Primer** | **Reverse Primer** |
| --- | --- | --- |
| Axin2 | ACCTACTCACTTCCATTCCCC | TCCTTTCCACATTCTCCCCAA |
| Bmp2 | GACTTCTTGAACTTGCCG | TGTTGCTTTTCTTCGCCTCC |
| Dmrt1 | TGCCAGTCTCTGTTAGCCAA | CTACAAACCTCAGCCGTGTG |
| Fgf9 | ATCTGACTCACACCCAACT | AGCAGCAGCCCGAAGACATT |
| Foxl2 | ATACGAATCAGAACGGAGCG | TCGGTGGGTTTTCTTGGC |
| Fst | AGAGAGAGAGAGGAGTCG | TCCACAAGTCAGAAGCAA |
| Fzd1 | ACACGCACATACACATACACCT | TTCCTCGCCAGCCACTGA |
| Gapdh | TCCTATCCTGGGAACCATCACC | TCTTTGGACCCGCCTCATTT |
| Hoxd13 | TGGGCTATGGCTACCACTTC | GACACTTCCTTGGCTCTTGC |
| Lef1 | GCTCCCAGGTTCTACAGATGGC | AAAATTCTCCGGTTCCCACTGTC |
| Lgr5 | CCTCTGGACCACAGGAAGT | AGACCATGACTGCGCTCTG |
| Oct4 | TGGCTGAGTGGGCTGTAAGG | CAAACCAGTTGCTCGGATGC |
| Rspo1 | GAAACTGGTCAGATGCTC | ACCCAGGATGCTTGCTAA |
| Sox9 | TCCTCCCTTTAGCCAACC | AGGCGTCTGGACTTAG |
| Wnt2b | GCAGTCAGCTAAATGGAGCAGA | AGCTGCTGGTTCCACTTGCTTC |
| Wnt4 | CGTGGGAGAAGTAATAAAAGAA | CTTAGGAACTGGAAGGCTGTG |
| Wnt5a | GCACTAACCCATGAGACATTG | TGTGAAGTTTAGGTGAACCG |

**Table 1. ChIP-qPCR primers**
