# Supplementary material for: CBX2 is required to stabilize the testis pathway by repressing Wnt signaling
Source: PLoS Genet. 2019 May 22;15(5):e1007895. doi: 10.1371/journal.pgen.1007895 (PMC6548405; doi:10.1371/journal.pgen.1007895)
Supplement: S2 Table — (DOCX) [file pgen.1007895.s011.docx]

**Table 2. Genotyping primers**

| **Genotype** | **Forward Primer** | **Primer mutant** | **Reverse Primer** |
| --- | --- | --- | --- |
| Cbx2 | GTAGCCAAGCCAGAGCTGAA | CCGCTTCCATTGCTCAGCGGT | ACCACAGGCCTCTTTGGTGT |
| Fgf9 | GCAAGGGAGGGGAGTTGGATATACC | GAAATCCAGTCCTGCAGTACAGCTGC | CAGCCCAAGCTTTCGCGAGCTCG |
| Wnt4 | CAACAACGAGGCTGGCAGG | CGCATTGTCTGAGTAGGTGTCATTC | CCCGCATGTGTGTCAAGATGG |
| XY | TGAAGCTTTTGGCTTTGAG | N/A | CCGCTGCCAAATTCTTTGG |
