# Supplementary material for: CBX2 is required to stabilize the testis pathway by repressing Wnt signaling
Source: PLoS Genet. 2019 May 22;15(5):e1007895. doi: 10.1371/journal.pgen.1007895 (PMC6548405; doi:10.1371/journal.pgen.1007895)
Supplement: S3 Table — (DOCX) [file pgen.1007895.s012.docx]

**Table 3. Immunofluorescence Antibodies**

| **Target** | **Catalog Number** | **Concentration** |
| --- | --- | --- |
| Foxl2 | Novus Biologicals NB100-1277 | 1:250 |
| Gata4 | Santa Cruz SC-1237 | 1:100 |
| PECAM1 | BD BioSciences | 1:250 |
| Sox9 | EMD Millipore AB5535 | 1:3000 |
| Sry | Gift from Dagmar Wilhelm | 1:100 |
