# Supplementary material for: CBX2 is required to stabilize the testis pathway by repressing Wnt signaling
Source: PLoS Genet. 2019 May 22;15(5):e1007895. doi: 10.1371/journal.pgen.1007895 (PMC6548405; doi:10.1371/journal.pgen.1007895)
Supplement: S4 Table — (DOCX) [file pgen.1007895.s013.docx]

**Table 4. ChIP Antibodies**

| **Target** | **Catalog Number** | **μl/IP** |
| --- | --- | --- |
| H3 | Active Motif 39763 | 3 |
| H3K27me3 | CST 9733S | 5 |
| H3K4me3 | Active Motif 39159 | 3 |
| Cbx2 | Bethyl A302-524A | 5 |
| IgG | CST 2729S | 3-5 |
